# Touching Base with Some Mediterranean Diseases of Interest from Paradigmatic Cases at the “Magna Graecia” University Unit of Infectious Diseases: A Didascalic Review

**DOI:** 10.3390/diagnostics13172832

**Published:** 2023-09-01

**Authors:** Ferdinando Carmelo Pio Lionello, Salvatore Rotundo, Gabriele Bruno, Gabriella Marino, Helen Linda Morrone, Paolo Fusco, Chiara Costa, Alessandro Russo, Enrico Maria Trecarichi, Anna Beltrame, Carlo Torti

**Affiliations:** 1 Department of Medical and Surgical Sciences, University “Magna Graecia”, 88100 Catanzaro, Italy; lionelloferdinando@gmail.com (F.C.P.L.); srotundo91@gmail.com (S.R.); bruga90@gmail.com (G.B.); gabriellamarino1@virgilio.it (G.M.); helen.morrone@gmail.com (H.L.M.); a.russo@unicz.it (A.R.); em.trecarichi@unicz.it (E.M.T.); torti@unicz.it (C.T.); 2 Unit of Infectious and Tropical Diseases, “Mater Domini” Teaching Hospital, 88100 Catanzaro, Italy; costach65@gmail.com; 3 College of Public Health, University of South Florida, Gainesville, FL 33620, USA; beltramea@usf.edu

**Keywords:** zoonoses, One Health, system immunology, vectors, infectious diseases, pathogens

## Abstract

Among infectious diseases, zoonoses are increasing in importance worldwide, especially in the Mediterranean region. We report herein some clinical cases from a third-level hospital in Calabria region (Southern Italy) and provide a narrative review of the most relevant features of these diseases from epidemiological and clinical perspectives. Further, the pathogenic mechanisms involved in zoonotic diseases are reviewed, focusing on the mechanisms used by pathogens to elude the immune system of the host. These topics are of particular concern for individuals with primary or acquired immunodeficiency (e.g., people living with HIV, transplant recipients, patients taking immunosuppressive drugs). From the present review, it appears that diagnostic innovations and the availability of more accurate methods, together with better monitoring of the incidence and prevalence of these infections, are urgently needed to improve interventions for better preparedness and response.

## 1. Introduction

### 1.1. Definition of Zoonoses and Main Components

Zoonoses are infectious diseases transmitted from animals to humans, either directly (through contact with skin, hair, blood, or secretions) or indirectly (through vectors or food ingestion) [1]. Vectors are living organisms that act as vehicles from animals to humans or vice versa [2]; these organisms must guarantee the survival, multiplication, and development of the pathogen [2]. Many vectors are ectothermic organisms or bloodsucking insects, which ingest disease-carrying microorganisms when they feed on the blood of an infected host (human or animal) [1]. These insects subsequently inoculate the pathogen into a new host during the next feeding. Climate, ecological (settlement of vectors in different geographical regions and proximity to the hosts), and socioeconomic factors (migration and exchange of goods) can influence pathogen transmission [2].

### 1.2. Transmission Mechanisms and Selected Zoonoses

Ticks are among the main vectors of zoonoses [3]. The *Ixodidae* family (hard ticks) and *Argasidae* (or soft ticks) are the most common in Europe. In Italy, there are thirty-six species grouped into seven genera [4]; the most prevalent is *Argas reflexus*, also known as the “pigeon tick”. Regarding impact on human health, the most widespread and relevant species both in Europe and Italy are *Ixodes ricinus* (known as the wood tick), *Rhipicephalus sanguineus* (dog tick), *Hyalomma marginatum,* and *Dermacentor reticulatus* [4].

The settlement of these arthropods depends on the presence of hosts [5]. Therefore, places such as stables, farms, and pastures are favored environments even if some species of wood tick bite wild animals and are common in wild environments (as happens, for example, with *Ixodes ricinus*) [5]. In the Mediterranean region, biodiversity hotspots and various outdoor activities (such as gardening, camping, hiking, and farming) are the main drivers of transmission. Moreover, the risk of tick bites is high in suburban and rural environments where several tick species are widespread [4].

*Ixodidae* require close attachment to the host and feed on its blood for several days to reproduce and develop [6]. The bite itself is not dangerous to humans, but the health risks depend on transmitted pathogens [5]; the injection of certain substances may play a role in the pathogenetic mechanisms [7]. For instance, tick saliva contains immune-modulating factors that create an advantageous environment for both tick feeding and pathogen transmission. In fact, cystatin sialostatin L (sialoL), which has been isolated from *Ixodes scapularis* saliva, inhibits the maturation of dendritic cells (DCs), antigen-specific stimulation, and proliferation of CD4+ T cells in the host organism [7]. Furthermore, other factors, such as antiplatelet agents, anti-vasodilators, and anticoagulants, are secreted with the saliva to facilitate feeding. Several studies have also confirmed the secretion of extracellular vesicles, such as exosomes [8], to evade the host response during feeding [9].

In addition to ticks, several other vectors may transmit zoonoses. These include fleas, lice, mosquitoes, and sandflies, although not all subspecies transmit pathogens and may be interchangeable with each other [2].

In this study, we focused on zoonoses with epidemiological relevance in the Mediterranean:

- Rickettsiosis;
- Ehrlichiosis;
- Borreliosis;
- Bartonellosis;
- Brucellosis;
- Leishmaniasis.

The causative pathogens are obligate intracellular organisms that require a host cell to complete their replication cycle and evade host defense mechanisms [10].

To evaluate the impact of zoonoses in the Mediterranean, we considered the latest data from Italian hospital discharge forms, which showed a reduction in hospitalizations for zoonoses such as brucellosis, rickettsiosis, bartonellosis, and borreliosis, although these latest updates were reported until 2013 [10].

Furthermore, a more recent study confirmed the high incidence of these zoonoses in Southern Italy [11]. From the serum samples of 135 subjects reporting tick bites, 62 (45.9%) tested positive for tick-borne pathogens such as *Rickettsia* spp. (21.5%) and *Borrelia* spp. (10.4%). However, positive antibody results may stem from previous bite exposure; therefore, the results of this study do not reflect current disease incidence, but rather pathogen circulation in the study area.

Although the actual incidence of zoonoses in the Mediterranean is largely unknown, their importance and likely increase underline the need for advanced solutions, not only for early diagnosis, but also for transmission containment and treatment. Therefore, this study reviews the clinical and epidemiological aspects of selected zoonoses, along with the main pathogenetic pathways of damage and long-term survival of these organisms in the host.

## 2. Zoonoses with Epidemiological Relevance in the Mediterranean Area

### 2.1. Rickettsiosis

Case Report #1: A 67-year-old woman affected by dyslipidemia, carotid atheromasia, and osteoporosis was admitted to the infectious diseases ward in August 2022. The clinical history began the previous two weeks when she had high fever (up to 40 °C) and maculo-papular rash. Interestingly, two brothers showed the same clinical pictures. In fact, all these people were “at risk” of acquiring rickettsial infection because they lived together in the same house in the countryside, owned pets, worked in the garden, and shared the same bench located in the park where they usually were sitting. Therefore, she and her brothers came to the emergency room of another hospital in Catanzaro and a chest radiography was performed which showed signs of interstitial pneumonia. Blood tests showed relative neutrophilia without leukocytosis and a C-reactive protein of 16.0 mg/L.

**Figure 1:**
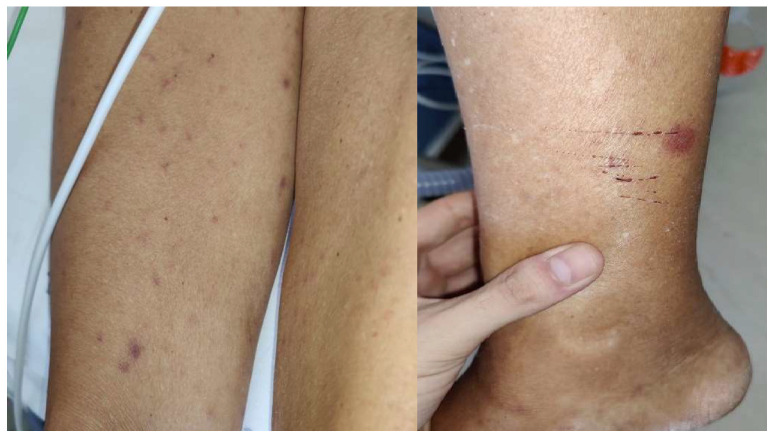
Rickettsial rash with spots which then turn into petechiae.

When she was transferred to our infectious and tropical diseases ward, she had asthenia, fever (38 °C), tachycardia (about 120 beats per minute), and tachypnea (breaths 26 per minute) with respiratory failure. Maculo-papular rash and tick bite were found at the physical examination (Figure 1).

Blood cultures were negative while serologic test for *R. conorii* was positive. During the hospital stay, the patient developed severe respiratory failure which needed high flow nasal cannula. Empirical antibiotic therapy with meropenem, linezolid, and levofloxacin was initially started. Based on the suspicion of rickettsial infection, high dose doxicicline (200 mg BID) was also started, with clinical improvement until recovery.

*Rickettsiae* are intracellular, obligate, Gram-negative bacteria that easily spread in the cytoplasm of host cells and are transmitted to the host (including humans) by arthropod vectors, such as “hard ticks” [12]. Most rickettsial species that are pathogenic to humans have arthropods (fleas, ticks, and lice) as vectors and reservoirs. Several species of wild animals, including tapirs, rodents, birds, and pets such as dogs, cats, and horses have also been considered to be potential sources of infection [13].

The species of *Rickettsia* are divided into four groups based on genomic classification:

1. Spotted fever (to which *R. rickettsii*, *R. conorii*, *R. parkeri*, and several others belong);
2. Typhus (*R. prowazekii* and *R. typhi*);
3. Ancestral (*R. bellii* and *R. canadensis*, even if not known to be pathogenic);
4. Transitional (*R. akari, R. australis, and R. felis*) [14].

Rickettsial diseases belong to the exanthematous fever group. Mediterranean Spotted Fever (MSF) is the most common tick-borne rickettsiosis in Mediterranean countries, particularly Italy [12]. Its main causative agent is *Rickettsia conorii* [14], although other species may also be involved, such as *R. monacensis*, *R. massiliae,* and *R. aeschlimannii* [12]. The incidence of rickettsiosis in Italy is of concern, given that the data may be underestimated due to diagnostic limitations (mainly linked to the difficulty in identifying the pathogen). The latest report indicates that 5989 cases occurred from 2001 to 2015, corresponding to an average annual incidence of 0.88 cases per 100,000 inhabitants (which decreased from 1.3 cases per 100,000 inhabitants in 2001 to 0.34 per 100,000 in 2015) [14]. The average annual hospitalization rate, standardized by age and sex (Standardized Hospitalization Ratio), was 1.36 cases per year for every 100,000 people, concentrated in groups aged over 24 years, with a higher risk for males than females (relative risk of 1.64). The mortality was 0.36% [14].

Higher standardized hospitalization rates were found on the Tyrrhenian coast, especially in Calabria, Sardinia, and Sicily, where rickettsiosis is a public health concern [14].

**Figure 2:**
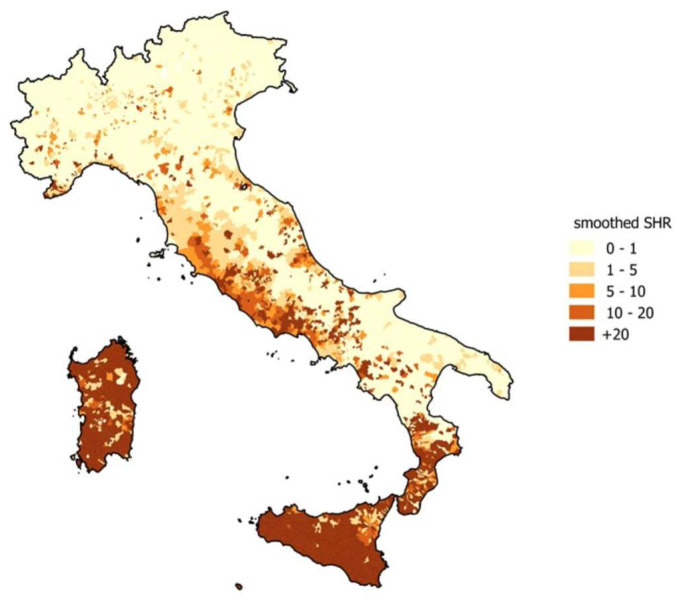
Standardized average hospitalization rate (number of hospitalized cases/100,000) for non-typhoid rickettsiosis from 2001 to 2015 in Italian municipalities [14].

As shown in Figure 2, four regions of Italy (Sardinia, Sicily, Calabria, and Lazio) have higher morbidity rates than the Italian average (11.9, 10, 4.7, and 3.9, respectively) [15].

Symptoms of MSF usually begin 4–10 days after tick bite [16]. The onset of the disease is usually abrupt, with severe headache, malaise, generalized myalgia (especially in the leg muscles), and fever [17] associated with a maculo-papular rash. The rash generally appears within five days of fever and is the main clinical feature; it mostly begins on the limbs and then spreads with a centripetal and caudocranial diffusion involving the palms and soles [18].

Many patients with MSF have an eschar or black necrotic scabbed lesion (*tache noire*) at the inoculation site, which is pathognomonic [17]. Renal, neurological, cardiac, phlebitis, and retinal complications can occur [19]. Risk factors for malignant MSF include advanced age, immunocompromised status, chronic alcoholism, glucose-6-phosphate dehydrogenase (G6PD) deficiency, prior prescription of an inappropriate antibiotic, and delay in treatment [20].

The clinical suspicion must be confirmed through serologic testing or polymerase chain reaction (PCR) testing, or by using special stains on a skin biopsy (Table 1). Acute and convalescent serology should be sent for patients clinically suspected of having rickettsial infection, using an indirect immunofluorescence assay (IFA) test. IgG antibodies typically became detectable 7 to 10 days after the onset of illness, and the optimal time to obtain a convalescent antibody titer is between 14 and 21 days after symptom onset [21,22]. A four-fold rise in IgG titers between acute and convalescent sera is indicative of seroconversion and recent illness. The use of IgM assays alone should be avoided for diagnosis [23] due to potential false-positive results resulting from cross-reactivity with lipopolysaccharides from bacterial pathogens [24]. Since the antibody response is not yet detectable during the first week of symptoms, therapy should not be delayed. The utility of PCR amplification of *R. rickettsii* DNA from blood is limited by its low sensitivity, particularly in early or mild disease, attributed to the low numbers of circulating rickettsial organisms in the blood [25]. While a positive PCR result for *Rickettsia* spp. in blood can be confirmatory, a negative result does not definitely rule out the diagnosis. If an eschar is present, the sensitivity of the PCR is higher [21]. Direct immunofluorescence testing or immunoperoxidase staining can be performed on a skin biopsy specimen. However, performing these tests is not recommended in patients who have received tetracycline (usually doxycycline) or chloramphenicol for more than 48 h, as the initial sensitivity of approximately 70% decreases after the initiation of therapy [20].

*Rickettsia* proliferates within vascular endothelial (EC) cells that line the small- and medium-sized blood vessels [27]. Endothelial damage activates several host defense mechanisms, including coagulation pathways and cytokine networks. In addition, it establishes transient immune dysregulation, characterized by a reduction in circulating peripheral CD4+ T lymphocytes and perivascular infiltration of CD4+ and CD8+ T lymphocytes, B cells, and macrophages [12]. These characteristics are clinically defined as “*rickettsial vasculitis*”. Thus, the pathogen proliferation mechanism and the host immune response are responsible for the characteristic maculo-papular rash due to blood vessel involvement.

Although cell-mediated immunity is thought to play an important role, host defense mechanisms are not yet fully understood [28]. *Rickettsia* preferentially infects the vascular system of susceptible mammals via mechanisms of oxidative damage mediated by reactive oxygen species (ROS) production [29]. ROS production is induced by the combination of tumor necrosis factor-α (TNF-*α*) and interferon-γ (IFN-γ), both of which act as host defenses [29]. Other possible mechanisms may include direct damage caused by complement systems or rickettsial phospholipase activity (phospholipase D or phospholipase A2) [12].

The complement system is activated in vivo during *Rickettsia* infection and may play a role in the immune response against this class of pathogens, enhancing the innate and adaptive responses [30]. It is a key serum-borne innate immune component consisting of a collection of soluble proteins activated by proteolysis, conformational changes, or macromolecular assembly, and is triggered by recognizing molecular markers on the surface of foreign cells. Furthermore, it can be triggered by the classical, lectin, and alternative pathways [31].

The classical complement pathway is stimulated by the deposition of antibodies on the surface (both IgM and IgG recruit the C1 complement complex) [30]. The lectin pathway is triggered by sugar-binding structures that recognize sugar molecules present on the surface of pathogenic organisms. Alternative pathways involve autocatalytic activation, triggered by regulatory proteins. These mechanisms converge in the generation of a C3 convertase enzyme that amplifies the proteolytic cascade, which includes three different effector mechanisms [31]:

1. Recruitment of C6–9 proteins to produce a lytic pore to kill the microorganism;
2. Opsonization from the deposition of C3b and C5b to improve phagocytic cell digestion;
3. Release of anaphylatoxins C3a and C5a, which recruit immune cells to areas of complement activation [31].

Although *Rickettsia* is inherently resistant to complement-mediated killing, a recent study [32] demonstrated the significant role of C1q (classical complement pathway protein) and pre-existing IgM in initiating a complement system in human and mouse serum. Mice without the C1q protein suffer reduced complement activation, a high tissue bacterial load, and a decrease in serum IFN-γ, suggesting the involvement of classical complement activation in response to *Rickettsia* [32].

As previously mentioned, the isolation of cystatin sialostatin L (sialoL) from *Ixodes scapularis* saliva inhibits the maturation of DCs and antigen-specific stimulation and proliferation of CD4+ T cells, thereby suppressing IL-9 production in Th9 cells (in vitro) [7].

IL-9 improves mast cell growth by inducing mastocytosis [33]. It is essential to initiate protective immune responses during parasitic infections, and mast cells are likely to be their most important innate sources. At the transcriptional level, the GATA-1 sequence regulates the expression of the Il-9 gene upon IgE-mediated mast cell activation [34]. Mast cell localization, as an interface between the host and the environment, and its contemporary expression of several Toll-like receptors (TLRs), make it an immediate early immune response center [35]. They perceive and boost by invading parasites, such as ticks, through their ability to rapidly release immune mediators, such as histamine and leukotrienes, and to serve as a primary source of cytokines, including TNF-α, IL-1β, and IL-9 [33].

### 2.2. Brucellosis

Case Report #2: A 23-year-old male patient who presented with fever every 5–6 days associated with chills and profuse sweating was admitted to our day-hospital service in June 2020, where among laboratory tests, a serological test for *Brucella* spp. (IgM) and blood RT PCR for *Brucella* spp. were positive. Abdominal ultrasound and echocardiography were also performed, excluding an infectious involvement of the examined organs. Full blood cell count, and renal and liver function tests, were normal. No abnormalities were found on physical examination. The result of microbiological tests and clinical suspicion led us to start therapy with doxycycline and gentamicin (for the first days of the hospital stay), continued for a total of six weeks. After antibiotic therapy, a significant improvement in clinical conditions was found, with fever and sweating disappearing and, at the last hospital check, blood RT PCR for *Brucella* spp. was negative.

Brucellosis (also known as “undulant fever”, “Mediterranean fever”, or “Malta fever”) is a zoonotic infection transmitted to humans from infected animals (mainly cattle, sheep, goats, camels, and pigs) [36] when consuming contaminated foods (such as unpasteurized dairy products) or from contact with infected tissues or liquids [37]. Brucellosis is a disease caused by a very small (0.6–2.0 × 0.3–0.5 μm), asporigenous, immobile, and Gram-negative bacillary or coccobacillary type without a capsule. It is the most common zoonosis worldwide and is a public health problem in many resource-limited settings [38]. To date, four *Brucella* pathogen species have been identified:

- *B. melitensis* (isolated from ruminants such as goats, sheep, or camels);
- *B. abortus* (isolated from livestock);
- *B. suis* (isolated from pigs);
- *B. canis* (isolated from dogs) [36,39].

Worldwide, most recorded cases in humans are *B. melitensis* infections. In December 2021, the European Food Safety Authority (EFSA) and the European Center for Disease Prevention and Control (ECDC) published an annual report on zoonoses, with data from 36 European countries in 2020 (twenty-seven EU Member States and nine non-members) [40]. According to this report, 128 cases of brucellosis were recorded throughout Europe, and the most affected countries were in the Mediterranean region; approximately 85% of cases in 2008 occurred in Greece, Italy, Portugal, and Spain [36]. Since 1996, as reported by the Italian Ministry of Health, a significant and continuous decline in the number of cases has been observed. The last incidence reported was 0.31% in 2012 (164 cases per 100,000 inhabitants) [36]. However, the actual incidence may be higher because of under-reporting.

*Brucellae* enter the human body via ingestion, inhalation, mucous membranes, or non-intact skin. The organisms are then taken up by polymorphonuclear cells or macrophages, where they can survive and replicate, evading the immune system [41], transfer through regional lymph nodes into circulation, and spread to reticuloendothelial organs, including the liver, spleen, and bone marrow [42]. The incubation period ranges from 2 to 4 weeks [35]. Often, systemic infections have an acute (<2 months), subacute (2–12 months), or chronic relapsing course (more than one year) with severe complications [43].

Fever is the most common feature of brucellosis, followed by osteoarticular involvement [44]. Malodorous perspiration is almost pathognomonic [41]. Other symptoms include headache, myalgia, back pain, cough, malaise, anorexia, fatigue, and weight loss [45]. Physical examination is generally non-specific [41] and the most common findings are hepatomegaly, splenomegaly, lymphadenopathy, osteoarticular manifestations, genitourinary complications, and neurological findings [44] (Table 2).

Clinically, Brucellosis does not always cause symptoms. The infection may be present for months without one noticing, as it can only be detected through blood tests [40]. Therefore, it may slowly evolve into a systemic infection, usually after a long incubation period.

A definitive diagnosis of brucellosis may be established through either of the following methods [46,47,48,49]: (i) isolation of the *Brucella* spp. organism from blood, body fluids (such as urine, cerebrospinal fluid, synovial fluid, and pleural fluid), or tissue samples (including bone marrow or liver biopsy); (ii) a four-fold or greater rise in *Brucella* spp. antibody titer between acute and convalescent phase serum specimens, collected at least two weeks apart.

A presumptive diagnosis of brucellosis can be made through either of the following methods [46]: (i) *Brucella* total antibody titer ≥1:160 as determined by the standard tube agglutination test (SAT) in a serum specimen obtained after the onset of symptoms; (ii) detection of *Brucella* spp. DNA in a clinical specimen by PCR.

*Brucella* spp. induce an active inflammatory reaction, penetrating the submucosa through the cells of the skin, conjunctiva, pharynx, or lungs. If the microorganism eludes the body’s defenses, the bacteria enter the blood (causing bacteremia) and spread to the spleen, liver, and marrow with the formation of granulomas [50].

“Classic” brucellosis is papular and pustular in form due to the onset of brucella allergy. It is more easily found in operators at occupational risk. Generally, the evolution of the disease occurs without clinical complications, but some forms can be severe.

Brucellosis in humans typically presents as high undulating fever, malaise, night sweats, and arthralgias with acute and subacute diseases [39]. Once the *Brucellae* are absorbed by local tissue lymphocytes, they enter systemic circulation through regional lymph nodes and thereby spread throughout the body, with particular tropism for the reticuloendothelial system. The incubation period tends to last 2–4 weeks from the start of symptoms and occasionally lasts up to several months. The fever pattern is variable, being either relapsing, mild, or protracted. Physical findings are variable and non-specific, with several studies having noted hepatomegaly, splenomegaly, and/or lymphadenopathy [39].

Chronic brucellosis can have several systemic implications that involve multiple organs in the host organism, causing hepatitis, encephalomyelitis, orchitis, arthritis, and endocarditis. Therefore, patients with brucellosis can be divided into two categories:

- Those with objective evidence of a *Brucella* infection (bacterium isolated from tissue, blood culture, or specific antibody titers) and/or focal complications (such as osteomyelitis, spondylitis, or uveitis) [39];
- Those with persistent symptoms in the absence of objective infection evidence (serological values or positive cultures); in this case, the symptoms may include general malaise, psychiatric disorders (depression, anxiety, and emotional lability), tremor or arthralgia, insomnia, or sexual disturbances [39].

The adhesion of *Brucella* to extracellular matrix (ECM) components is the first step in triggering infection [51]. *Brucella* binds to host epithelial cells through molecules containing sialic acid and/or sulfated residues and through components of the ECM (such as fibronectin, collagen, and vitronectin) [52]. A recent study [51] reported the involvement of specific adhesins, such as the *BtaF* trimeric autotransporter, in this interaction. In this study, *BtaF* was identified as a potential solution for a new vaccine against brucellosis. Indeed, this protein induces a specific humoral immune response in mice, both locally and systemically, as well as a mixed T helper response (with Th1 phenotype differentiation) and central memory CD4+ T cells [51].

*Brucella* interacts with the host’s epithelial cells across the intestinal mucosal epithelium layer, replicates inside phagocytic cells, and triggers the production of pro-inflammatory cytokines such as IL-1, IL-6, IL-7, and IL-8 [53]. It has strong tropism for macrophages, DCs, placental trophoblasts, and a wide variety of cell types, including microglia, fibroblasts, and epithelial and endothelial cells [52].

Cytokines play an important role in protecting against brucellosis by mediating both adaptive and innate immune responses. B cells and macrophages release the cytokine IL-12, which differentiates Th1 cells and releases IL-1β, and IL-6, and interferon γ (IFN-γ), which is amplified by TNF-α [54]. This increases bactericidal capacity and enables cytotoxic T lymphocyte (CTL) action. Contemporarily, after *Brucella* infection, neutrophils and macrophages infiltrate the spleen and increase the expression of IL-2, IL-10, and IFN-γ while reducing IL-4, which is involved in Th2 response [50,55].

Th1 involvement requires functional TLR9/MyD88/IL-12p35 signaling pathways to eradicate *Brucella* from the spleen [56]. Their absence increases the susceptibility to infection [57].

*Brucellae*, like other intracellular pathogens, modify the innate immune response to replicate and persist long-term. The failure of long-term protection against *Brucella* is the result of this decreased adaptive immune response efficiency, which is linked in part to the attenuated innate immune response [57].

To date, there is no significant progress in *Brucella* vaccination, except in China, where an attenuated strain (*Brucella suis* S2) is used for swine vaccination [58]. A recent study [59] evaluated the vaccine potential of a trimethyl chitosan (TMC) nanoparticle formulation of urease (TMC/urease) against brucellosis in mice. In this study, immunization with TMC/urease nanoparticles induced highly specific IgG production. In particular, the study reported different immunological response profiles depending on the route of administration; intraperitoneal administration of both urease alone and TMC/urease nanoparticles induced a mixed Th1–Th2 immune response, whereas oral administration induced a mixed Th1–Th17 immune response [59]. These results confirm that the nanoparticle delivery system may be a potential solution for *Brucella* immunization.

### 2.3. Bartonellosis

Case Report #3: A 42-year-old Caucasian male (weight: 84 kg, height: 178 cm) came to our observation in July 2013 complaining about asthenia associated with a right latero-cervical lymphadenomegaly, which began two years earlier. The patient, along with these symptoms, had never reported fever. He was resident in a rural area, living in contact with several animals (in particular birds and cats) and worked as a volunteer in a human hospital. The patient reported allergy to diclofenac, cipressaceae, and parietaria plants. He underwent two interventions for inguinal hernia, the first at the age of 11 years and the second at the age of 34. In 2004, he suffered from kidney stones. In 2004 he had toxoplasmosis. In July and August 2014 he was referred twice to the Unit of Neurology because of a previous diagnosis of epilepsy treated with levetiracetam and topiramate from 2002 to 2007. Upon admission to the neurology ward, serological tests for several agents were performed (*T. gondii, Cytomegalovirus, Epstein-Barr virus, Brucella*, HBV, HCV, HIV, *T. pallidum, Salmonella* spp.), and all were negative. Full blood cell count was normal. Lymph node ultrasound (US) documented the presence of numerous lymph nodes at submandibular, lateral cervical, axillary, and groin regions with maximum diameter of about 2.5 cm. So, we decided to perform both serology tests and RT PCR for *Bartonella* spp., for the suspicion of CSD. The result of a positive RT PCR for *B. henselae* led us to start therapy with 100 mg of doxycycline, twice daily, per os for ten days. Then, the patient discontinued treatment for epigastralgia. After 9 days, he restarted doxycycline 100 mg, twice daily, per os. We suggested that he, in order to avoid further epigastralgia, take a gastroprotective therapy, so he was able to continue an appropriate treatment for two weeks. This therapy was stopped after RT PCR found *B. henselae* DNA to be undetectable. After antibiotic therapy, there was a significant reduction in lymphadenopathy and fatigue, and asthenia recovered. At the last outpatient consultation, in June 2014, just a small left later cervical node was evident, followed by complete regression in January 2015 [60].

The *Bartonella* genus includes Gram-negative intracellular bacteria that usually live within the red blood cells and endothelial cells. Humans acquire the infection through a vector. *B. quintana* is transmitted through fleas and head lice (*Pediculus humanus var corporis*), *B. bacilliformis* through the sting of certain species of sand fly [61], and *B. henselae* (the main zoonotic species of *Bartonella*) through cats (main reservoir) [62]. In this case, the feline contracts the infection from a flea or tick bite or through flea feces, and consequently transmits it to humans through scratches or bites [62].

The latest data show that, in Italy, the average incidence rate of hospitalizations (number of cases/1,000,000) was 1.85/1,000,000 from 2009 to 2013, calculated from the Italian resident population by age, sex, and province [63].

The main pathogenic species are *B. quintana* [64]*, B. henselae* [65], and *B. bacilliformis* [66].

*B*. *quintana* causes a broad repertoire of diseases, including classic trench fever, chronic bacteremia, bacillary angiomatosis, endocarditis, and chronic lymphadenopathy [67]. The name “trench fever” was given because the disease was associated with both Allied and German troops during World War I [68] and is characterized by attacks of fever that last 1–3 days and are associated with headache, shin pain, and dizziness; the attacks recur every 4–6 days [69]. The term “quintan fever” refers to 5-day recurrences; usually, each succeeding attack is less severe than its predecessor. Trench fever often causes prolonged disability. The illness persists for 4–6 weeks [70]. At the end of the 20th century, *B. quintana* re-emerged as an agent of disease, often among homeless alcoholic men in Europe and North America [67,71], living in unsanitary conditions [72], and caused “urban trench fever”. *B. quintana* often manifests as bacteremia, endocarditis, or bacillary angiomatosis (BA) [64], especially in immunocompromised hosts [67].

*B. bacilliformis* infection in humans typically takes a biphasic course, resulting in Oroya fever, an acute life-threatening hemolytic anemia that, in untreated patients, may subsequently progress to include vascular proliferative skin lesions, termed verruga peruana [66].

*B. henselae* infection presents varying clinical symptoms ranging from lymphadenopathy to systemic diseases. The most common clinical form is cat-scratch disease (CSD) (89%) followed by endocarditis (9%). Disseminated forms (2%) mainly present as BA or peliosis hepatis in solid organ transplant recipients [65,73]. Cats may infect humans either directly through scratches and bites, or indirectly via cat flea (*Ctenocephalides felis*), which is an arthropod vector [74]. Patients with CSD develop regional lymphadenopathy [73]. Three to five days after exposure, a papule appears on the skin. The papule progresses through a vesicular and crusty stage within two or three days, and approximately two weeks later lymphadenopathy occurs [75]. The most commonly involved sites are the axillary, epitrochlear, cervical, supraclavicular, and submandibular regions [76]. Lymphadenitis is usually associated with systemic symptoms such as generalized aching, malaise, anorexia, fever, nausea, and abdominal pain [76], although many patients have a gradual resolution within several months [73]. Some develop several clinical abnormalities, such as Parinaud’s oculoglandular syndrome, encephalopathy, myelitis, peripheral neuropathy, neuroretinitis, granulomatous hepatitis, granulomatous splenitis, erythema nodosum, and osteolytic bone lesions [73]. In immunocompromised hosts, infections may be disseminated and associated with peliosis in the liver and spleen, particular in HIV-infected patients who develop BA, mostly located on the skin [65,77]; the lesions are clinically indistinguishable from those of Kaposi’s sarcoma [73].

Bartonellosis tends to be mild and self-limiting but can sometimes become severe [65]. The severity of the symptoms depends on the host’s immune status. In immunocompetent patients, the course is self-limiting and resolves within a few weeks. The risk is greater in immunocompromised patients whose clinical course can involve chronic skin rashes (Peruvian warts), trench fever [78], BA [64], persistent bacteremia [64], and endocarditis [65]; however, there have been reports of systemic complications, even in immunocompetent patients [65].

*Bartonella* spp. are intracellular pathogens that trigger a Th1-mediated immune response in the host [79]. This pathway involves IFN-γ and TNF-α as key factors, which activate macrophages and release nitric oxide to eliminate the bacteria [79].

Many other factors are involved in the pathogenesis of *Bartonella* [80]. Some of these are reported in Table 3.

Similar to *Rickettsia* spp., *Bartonella* spp. show tropism for endothelial cells that persist and replicate [80]. A compromised immune system allows for abnormal progression and proliferation of vascular endothelial cells [65]. Trigger factors, such as adhesin, deformin, and outer membrane protein (OMP) are involved in this process through undetermined mechanisms, as described in Table 3 [80].

Furthermore, a recent study [79] reported that the liver was the most affected organ in *B. henselae*-infected *Ahnak*-knockout (KO) mice, with more granulomatous lesions and inflammatory cytokine levels compared to the liver tissues of wild-type. Neuroblast differentiation-associated protein (AHNAK) is the largest protein involved in muscular regeneration, formation of cytoskeletal structure, calcium homeostasis, and other biological processes such as obesity and cellular adipogenesis [79]. It is also a tumor suppressor protein that prevents the development of breast and lung cancer [81]. The *Ahnak*-KO mice demonstrated low levels of IFN-γ and IL-4 against *Bartonella* infection, confirming a Th1-mediated immune response in the host [81]. This suggests that AHNAK is necessary to induce a Th1 immune response against *Bartonella*.

Since *Bartonella* spp. are intracellular bacteria, their isolation through culture is very difficult and slow, requiring serological tests for diagnostic purpose, often affected by false negative results. Some case series indicated the utility of molecular identification and quantification of *Bartonellae* spp. infections with species-specific RT PCR, able to distinguish *B. quintana* from *B. henselae* strains by melting temperature [60].

### 2.4. Ehrlichiosis

The family *Anaplasmataceae* contains several species of intracellular, obligate, and Gram-negative bacteria that include the genera *Anaplasma* and *Ehrlichia*. *Ehrlichiae* do not possess flagellin, peptidoglycan, or lipopolysaccharides (LPS) and can be transmitted by ticks [82]; their pathogenicity in humans did not emerge until 1987, when the first human case of monocytic ehrlichiosis was reported. *Ehrlichia chaffeensis* was isolated in 1991 in the United States, and human granulocytic ehrlichiosis was first described in 1994 [82]. In 2001, based on the sequencing of the rRNA 16S gene, *Ehrlichiae* was included in the genus *Anaplasma* and divided into three species [82]:

- Neoehrlichia: *Candidatus* N. mikurensis and N. lotoris;
- Ehrlichia: *E. minasensis*, *E. ruminantium*, *E. canis*, *E. muris* subsp *muris*, *E. muris* subsp *eauclairensis*, *E. chaffeensis*, the Panola Mountain Ehrlichia, and *E. ewingii*;
- Anaplasma: *A. phagocytophilum*, *A. marginale*, *A. centrale*, *A. ovis*, *A. platys*, *A. bovis*, *A. capra*, and *Aegyptianella pullorum* [82].

In 2005, the highest number of ehrlichiosis cases occurred in the United States, where it is still considered an emerging infectious disease [83,84]. This included 471 cases of human monocytic ehrlichiosis (HME), for a total of 2396 cases since 1986, and 700 cases of human granulocytic anaplasmosis (HGA), with 2963 cases since 1994 [84].

Some cases of HGA have been diagnosed in the Italian islands of Sicily and Sardinia and in northeastern Italy; in the Alpine area, antibody positivity was observed in individuals highly exposed to tick bites [85].

HME can occur asymptomatically. In most cases, 1 to 2 weeks following exposure to an infecting tick, patients experience malaise, low-back pain, or gastrointestinal symptoms, or may develop sudden onset of fever [77]. Approximately one-third of adult patients and more than half of children infected with HME report a maculo-papular rash in the early stages, which can be petechial in the late stages [86]; the rash may involve the extremities, trunk, face, or, rarely, the palms and soles [77]. In a recent Polish study, conducted on 183 patients hospitalized for medical service (between the 3rd and 71st day after tick bite) DNA of *A. phagocytophilum,* from PCR analysis, was found in 7.1% (13/183) of blood samples and 8.2% (15/183) of skin biopsies, confirming that 8.1% of erythema migrans rashes after a tick bite had genetic material of *A. phagocytophilum* [87]. The findings were subsequently confirmed by the same research group, which showed that skin lesions resembling erythema migrans were reported in 19 (15.6%) of 120 patients enrolled and affected by HGA [88].

To diagnose HME or HGA through serologic testing, two blood samples are needed: one should be collected upon presentation (referred to as an acute sample) and the second should be obtained two to four weeks after sending the initial antibody test (referred to as a convalescent sample). According to CDC guidance, a definitive diagnosis requires at least a four-fold change in the IgG titer between the acute and convalescent stages [89]. For confirmation of an infection, most laboratories stipulate that at least one of the IgG titers should measure between 1:64 to 1:80 [22].

Other manifestations include cough, pharyngitis, lymphadenopathy, diarrhea, vomiting, abdominal pain, and changes in mental status [77]. If untreated or if treatment is delayed, severe complications may occur, including adult respiratory distress syndrome (ARDS), disseminated intravascular coagulation-like syndrome (DIC), central nervous system involvement, and renal failure [86]. Immunocompromised patients have the highest risk of severe outcomes [86].

In most patients, HGA shows flu-like symptoms (fever, myalgia, arthralgia, and headache) [90]. The symptoms and signs range from asymptomatic to fatal [91]. Most symptomatic patients report exposure to ticks 1–2 weeks before the onset of symptoms [92]. Local skin reactions at the site of tick bite have not been described, and the presence of skin rashes has been reported only occasionally [91]. In a few patients, it is possible to observe pulmonary manifestations such as dry cough and pneumonia; gastrointestinal manifestations include nausea, vomiting, diarrhea, and abdominal pain, and, more rarely, hepatomegaly, splenomegaly, or both [90]. Complications include septic or toxic shock-like syndrome, respiratory insufficiency, rhabdomyolysis, pancarditis, acute renal failure, hemorrhage, and neurological diseases [92].

Both HGA and HME share many clinical and laboratory manifestations, including fever, headache, myalgia, malaise, thrombocytopenia, leukopenia, and indices of hepatic injury (Table 4) [84].

Clinical complications are rare (Table 5), although they may present early within days of onset or, sometimes, late and persist for long periods, even in the absence of active infection [84].

However, the clinical complications of HME and HGA differ [83]. Patients affected by HME can develop fulminant shock-septic syndrome, primarily those with a marked immunocompromised state. Instead, clinical complications in HGA patients are more common, including peripheral neuropathies (brachial plexopathy, demyelinating polyneuropathy, isolated facial paralysis) that persist for weeks to months [83]. Conversely, respiratory distress syndrome may occur in both HME and HGA. Deaths caused by HME involve approximately 3% of all infections, especially in immunocompromised individuals with respiratory distress syndrome, hepatitis, or opportunistic nosocomial infections. Mortality is lower in HGA (approximately 0.7%) due to complications from opportunistic infections [84].

*Ehrlichiae* show strong tropism for mononuclear phagocytes, both granulocytes (human granulocytic anaplasmosis and *Ehrlichia ewingii* ehrlichiosis) [93], and monocytes (human monocytic ehrlichiosis) [92], for which they have developed survival and replication mechanisms to evade host immunity. Unlike *Rickettsia*, which escapes the endocytic vacuole to survive freely in the cell cytoplasm [12], *Ehrlichiae* replicate within modified vacuoles and form microcolonies, known as morulae [94,95]. Morulae are microscopically observable intracytoplasmic inclusions resulting from the development, proliferation, and division of bacterial colonies [95].

*Ehrlichia* infection activates T cell-mediated immunity [95]; DCs produce IL-12, CD4 T cells are activated, and Th1 effector cells are differentiated [96]. Natural killer T cells (NKT cells) are the second CD4 T cell population that may be involved in host resistance to *Ehrlichia* species. In this context, the role of CD4+ T cells has raised more questions over the last few years. These cells can also increase IFN-γ production during infection [96].

Knockout mouse strains with silenced immune genes have provided considerable information on this topic. In a study performed on β2M knockout mice (CD8+ T cell-deficient), the survival rate of CD4+ T cells increased [95]. Furthermore, T helper cell depletion increased the number of deaths in mice [96].

The host humoral response against *Ehrlichia* occurs, in part, during the extracellular phase, which blocks the entry of bacteria or interferes with the mechanisms linked to Fcγ receptors (FcγR) [97]. It also occurs through other partially defined mechanisms mediated by intracellular and extracellular antibodies, with specific immune complexes or intracellular receptor formation, such as tripartite motif-containing (TRIM21) receptor [97].

Understanding protective immune mechanisms is necessary to develop vaccines against *Ehrlichia* or other intracellular pathogens. Several studies [98] have shown that specific monoclonal antibodies against the immune serum or outer membrane proteins can protect mice with severe combined immunodeficiency from fatal *Ehrlichia* infection, even if administered after the infection has developed. Furthermore, passive administration of antisera and antibody administration protected mice against lethal infection [98]. This study demonstrated the involvement of intracellular and extracellular antibodies in *Ehrlichia* pathogenesis, which may open future research opportunities.

### 2.5. Lyme Borreliosis

**Figure 3:**
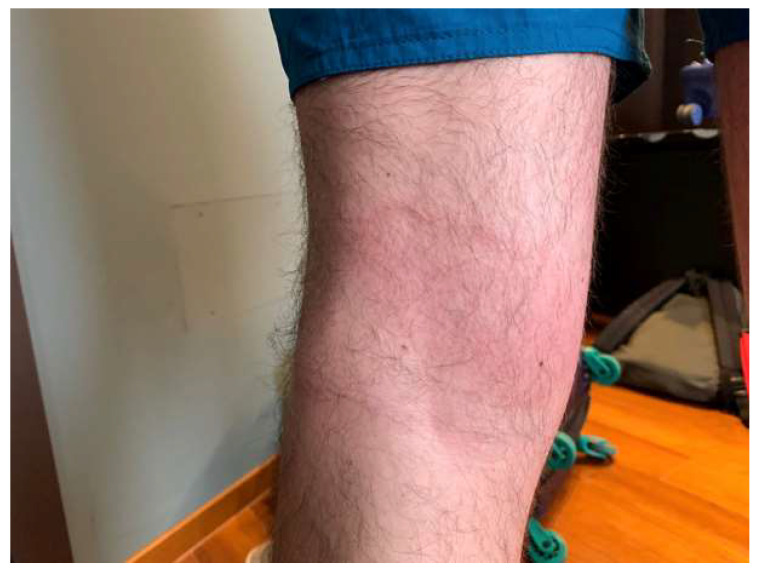
Red macula typical of Lyme disease in a patient.

Case Report #4: A 31-year-old male patient reported an insect bite which occurred in August 2022 while staying in a wooded area. After a few days, he noted the appearance of a macular rash in the popliteal cavity, which became bigger in the following days (Figure 3).

He started therapy with corticosteroids, antihistamines, and amoxicillin 1 g TID (stopped after four days due to diarrhea). Therefore, he came to our clinic. He did not report fever or pain and at clinical examination no rash elsewhere was observed. Serologies for several infections were performed and all were negative except for serology (IgM) for *Borrelia*, which was positive. No other symptoms or signs of the disease were reported. Treatment with 100 mg of doxicicline BID was prescribed for ten days, resulting in the complete resolution of the rash.

Lyme borreliosis is a multisystemic disease caused by a pathogenic species of *Borrelia*, which is transmitted to humans through an infected tick bite (prevalently the genus *Ixodes*) [99]. It is caused by spiral-shaped motile bacteria with two cell membranes similar in structure to Gram-negative bacteria, and flagella located specifically between them, rather than on the surface. Borrelia are 8–30 μm long and approximately 0.2 μ wide [100].

The main species in Europe are *B. burgdorferi*, *B. afzelii*, and *B. garinii*, while other *Borrelia* species have been isolated from humans, including *B. mayonii, B. spielmanii, B. bavariensis, B. valaisiana, B. lusitaniae*, and *B. bissettii* [99]. Among these, *B. burgdorferi* is the predominant infectious strain in North America, although it is less common in Europe. In Asia and Europe, infection is caused primarily by either *B. afzelii* or *B. garinii*, and infrequently by *B. spielmanii* or *B. bavariensis* [101].

Lyme disease is named after an unusual juvenile arthritis epidemic observed in 1975 among the inhabitants of Old Lyme County, Connecticut (United States) [101]. In 1977, several studies demonstrated a correlation between tick bites and the onset of skin erythema and joint symptoms [102]. A disease with similar symptoms was observed almost a century earlier in Europe.

In 1982, Burgdorfer and Barbour isolated a spirochete (later named *B. burgdorferi*) from the intestine of the *Ixodes* tick genus, and two years later, the same bacterium was isolated from the skin lesions, blood [103], and cerebrospinal fluid (CSF) of patients with the disease [104]. Consequently, the *Ixodes scapularis* tick was identified as the vector responsible for the infectious disease [100].

In Europe, an incidence of 22.4/100,000 person-years for Lyme borreliosis has been reported [105,106]. In Italy, the first clinical case was reported in 1983 in Genoa, and the germ was first isolated in Trieste in 1987 [105]. The incidence reported ranged from 0.02 to 0.001 new cases per 100,000 inhabitants from 2001 to 2005 [107,108]. The incidence of the disease has increased in Liguria, Friuli Venezia Giulia, Trentino Alto Adige, and Veneto. A recent study showed that, in Veneto, 1259 cases of Lyme borreliosis were diagnosed in the period 2006–2019, with an annual incidence of 1.916/100,000 inhabitants [109].

*B. burgdorferi* is transmitted from the tick to the host within 72 h after the tick bite [110]. *Erythema migrans* is the hallmark of Lyme borreliosis, a skin manifestation that begins as a red macule or papule, often with central clearing [111]. The lesion develops days to weeks later at the site of the infected tick bite [112], and is often accompanied by malaise, fatigue, headache, arthralgia, myalgia, fever, and regional lymphadenopathy [113]. Patients may also present with systemic symptoms without skin lesions [114].

If immediately treated, Lyme borreliosis responds well to oral antibiotics; left untreated, the spirochete can disseminate and cause severe illness for months or years after infection [16], accompanied by systemic symptoms such as severe headache, mild neck stiffness, fever, chills, migratory musculoskeletal pain, arthralgias, profound malaise, and fatigue [111].

If the disease follows this natural course (untreated), several months after the acute onset of symptoms, the following impacts on the patients should be noted:

- Joint involvement with swelling and pain, particularly in the large joints; the knee joint is affected with a pattern of oligoarticular arthritis that can evolve to severe chronic arthritic lesions [111].
- Cardiac involvement with acute onset of intermittent atrioventricular heart block, which is sometimes associated with clinical evidence of myopericarditis [112].
- Neurological involvement including meningitis, unilateral facial palsy, other cranial neuritis, and radiculitis may occur. Rare manifestations of the central nervous system include encephalomyelitis and chronic meningitis [111].
- *Borrelial lymphocytoma* is a rare skin manifestation (mainly seen in Europe) that presents as a solitary swelling most frequently located on the ear lobe and in the region of the areola mammae [112].
- *Acrodermatitis chronica atrophicans* is a skin manifestation exclusively observed in Europe, often seen on acral parts of the body and on the extensor part of hands or feet, and initially is usually unilateral; later, it may become more or less symmetrical with thin and wrinkled skin [112].

Clinical findings can be complemented by both serological testing and PCR [115]. The serological diagnosis approach often begins with a sensitive enzyme immunoassay, such as a whole cell-based enzyme-linked immunosorbent assay (ELISA). If the initial immunoassay yields a positive result, a more specific serologic test, such as the Western blot, is conducted. In the United States, a positive IgM Western blot within the initial 30 days of symptoms (early Lyme disease) requires identifying a minimum of two specific bands among 23, 39, and 41 kD. Subsequently, a positive IgG Western blot is affirmed when a minimum of five IgG positive bands (among 18, 21, 28, 30, 39, 41, 45, 58, 66, or 93 kD) are detected. However, in Europe, a universally applicable set of criteria with adequate sensitivity and specificity is currently lacking. This is primarily because, unlike North America, Europe hosts several *Borrelia* spp. (*B. afzelii, B. garinii,* and *B. burgdorferi*), each exhibiting considerable diversity in terms of the antigens crucial for serodiagnosis [115]. IgM antibodies against *B. burgdorferi* typically emerge within one to two weeks, and IgG antibodies usually appear within two to six weeks following early localized Lyme disease onset. However, only 20 to 40 percent of patients present as seropositive at the onset of the disease. Consequently, prompt initiation of therapy is advised if clinical suspicion is high. PCR has been employed in clinical setting to detect *B. burgdorferi* DNA in cerebrospinal fluid (CSF) or synovial fluid samples [116,117]. PCR results for *B. burgdorferi* in synovial fluid often prove positive prior to antibiotic treatment [118]. However, there are significant limitations associated with PCR use [119]: (i) the accuracy of PCR is highly reliant on proper sample collection, storage, and the assay technique; (ii) false-positive results are frequent, particularly in CSF PCR, which is more likely in seronegative patients for Lyme disease; and (iii) CSF PCR exhibits low sensitivity [120]. Thus, it is important to recognize that a negative PCR result does not rule out neurologic Lyme disease or Lyme arthritis. Four promising laboratory investigation methods are currently gathering attention for their potential to enhance diagnostics in Lyme disease [115]. Firstly, interferon gamma release assays (IGRAs) capitalize on T cell activation triggered by *B. burgdorferi* antigens, resulting in interferon gamma release [115,121]. These assays not only hold promise for early detection during the serologic window period, but also for distinguishing active infections from treated ones [122]. Secondly, in the realm of neuroinflammation, the chemokine CXCL13 has emerged as a pertinent marker [123]. Elevated CXCL13 levels exhibit a strong association with Lyme meningitis, suggesting its diagnostic value [121]. However, challenges persist in differentiating CXCL13 elevation due to Lyme disease from other conditions [124]. Thirdly, innovative metabolomics and proteomics approaches have unveiled potential biomarkers for early Lyme borreliosis [125]. Despite their complexity, these techniques offer insights into the disease’s underlying mechanisms. Lastly, metagenomics presents an unbiased means to detect *B. burgdorferi* genomic DNA in plasma or CSF samples [126]. Paired with targeted deep sequencing, this method has the potential for sensitive infection detection and characterization. However, before these methods can be incorporated into routine clinical management, it is crucial to undertake standardization efforts and rigorous validation. These diagnostic tools should be considered complementary to serology, enhancing our diagnostic capabilities while ensuring their practicality and precision within clinical settings [127].

Ticks provide *Borrelia* with the first line of defense against the host’s immune response, allowing it to survive and replicate in the host organism through several adaptation mechanisms [128]. Spirochete motility and chemotaxis are key to local and systemic dissemination, as they adhere to the vascular wall or extravascular tissue components, which facilitates the persistent colonization of diverse tissue sites [129]. In Lyme borreliosis, *Borrelia* has several mechanisms for long-term survival in the host and to maintain spirochetes in the tick–vertebrate infection cycle, altering the encoding of multiple adhesins and the correct functioning of mammalian host complement systems [128].

Humoral immunity is critical for eliminating *Borrelia*. Several studies [130] have successfully demonstrated that CD8+ CD60+ T cell numbers were significantly increased, almost four-fold, in patients infected with *Borrelia* compared to those in uninfected patients. CD60+ T cells are involved in T cell activation in both healthy and diseased states, including atopy, cancer, and autoimmunity. These results confirm that CD8+ CD60+ T lymphocytes play an important role in the immune response and that IgE is involved in anti-*B. burgdorferi* immunity [130].

Regarding vaccines, some prophylactic strategies have been developed. The antigens chosen in a recent study [131] interfered with tick feeding and/or *B. burgdorferi* transmission. *B. burgdorferi*’s outer surface protein C (OspC) has long been considered a Lyme borreliosis vaccine candidate. OspC production is induced during blood feeding, whereas spirochetes reside in the tick midgut [132]. Antibody-mediated immunity against OspC can prevent its dissemination to the host during early infection. However, OspC is strain specific, and over 30 distinct OspC types have been identified worldwide [131]. Therefore, DNA vaccination may be an alternative vaccine platform for inducing both humoral and cellular host immune responses [133]. Thus, DNA vaccination may be considered as a new candidate for tick vaccines. Indeed, using the recombinant OspC vaccine (rOspC) as a positive control, the DNA vaccine was found to be effective, as both the rOspC and OspC DNA vaccines were able to induce a robust IgG response [133].

### 2.6. Leishmaniasis

**Figure 4:**
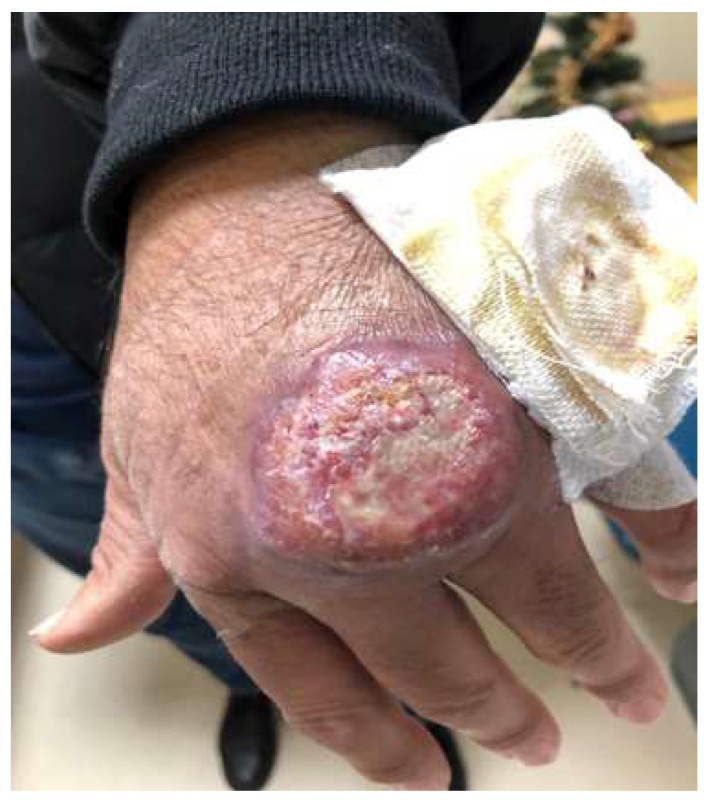
Red thickening of the skin (papule) typical of cutaneous leishmaniasis, which slowly enlarges and ulcerates.

Case Report #5: We report a case of a 52-year-old male patient (Figure 4), who presented a papule on the back of the hand that was present for about two years. The skin lesion was initially small and hyperemic, then progressively increased in size, and a well-defined and painless open ulceration, with a raised reddish border appeared. After several non-diagnostic biopsies, he was admitted to our day-hospital service in September 2020, in which scraping of the cutaneous lesion was performed, demonstrating the parasite (*L. infantum species*). In addition, serology (*Leishmania* IgG) was positive. An HIV test was negative and protein electrophoresis showed a monoclonal gammopathy. Physical examination failed to demonstrate enlarged lymph nodes. Abdominal ultrasound was also performed, excluding splenomegaly. The lesion was >1 cm, so the patient took a test dose of liposomal amphotericin B without side effects and was subsequently admitted to the ward, where he was successfully treated with intravenous liposomal amphotericin B (dosing schedule was 3 mg/kg/day on days 1–5, 14, and 21 with a total dose of 21 mg/kg).

Leishmaniasis is an infectious disease caused by protozoa of the genus *Leishmania*, an obligate intracellular parasite of the reticuloendothelial system in humans and other mammals [134]. It is transmitted to humans by a biological vector, *Phlebotomus* (hematophagous insect 2–3 mm in size, belonging to the genus *Phlebotomus*) and other similar sand flies [135]. The infection is caused by a sting from a phlebotomist, which has previously stung a parasite-infected animal [136]. Leishmaniasis primarily affects dogs, which in our area represent the main “reservoir” of the parasite, but it also affects cats and wild rodents, as well as humans, mainly the elderly, children, and immunosuppressed [137]. Localized infection (near the insect bite) leads to cutaneous leishmaniasis (CL); if the parasite overcomes the skin defense barrier, the patient may develop mucocutaneous leishmaniasis (ML) or visceral leishmaniasis (VL) [138].

Leishmaniasis is considered by the World Health Organization (WHO) as one of the six neglected tropical diseases (NTDs) [139]. It is endemic in temperate, tropical, and subtropical areas of the world, and throughout the Mediterranean basin. It is prevalent in Africa, Central and South America, Asia, and the Mediterranean region. The disease affects approximately two million people per year [137]. WHO recognizes that the areas in which leishmaniasis is endemic, as well as the number of cases recorded worldwide, have expanded considerably since 1993 [139]. The disease is now endemic in 88 countries on five continents, with a total of 12 million infected people and over 350 million people at risk [140].

About 1.5–2 million new cases are estimated annually, and 600,000 are officially declared. Nearly 60,000 people died in 2001. The majority of VL cases (around 90%) occur in five countries: Bangladesh, Brazil, India, Nepal, and Sudan [138]. The same percentage of ML occurs in Bolivia, Brazil, and Peru, while the greatest incidence of CL occurs in Afghanistan, Brazil, Iran, Peru, Saudi Arabia, and Syria, with at least 1–1.5 million new cases recorded each year [140].

In the 1990s, a leishmaniasis epidemic devastated Sudan, with more than 100,000 deaths. In 2004, another epidemic of CL occurred in Kabul, Afghanistan, with over 200,000 cases, of which 67,500 were in the capital alone [140].

Throughout the Mediterranean area, the disease re-emerged, with an increase in cases throughout the 1990s [141]. In Italy, data from the Higher Institute of Health showed that the annual incidence at the beginning of the 2000s was approximately 200 cases, although many regions were under-notified, and in March 2004, the disease was widespread among immunosuppressed people. Active surveillance programs have been established in the Campania, Sicily, and Liguria regions [141].

In the macro-region of Northern Italy, the average percentage of serum incidence in 2017 was 14.21% among a total of 24,716 tests, of which 3513 were positive, with a cutoff value of 1:40 [141].

In the macro-region of Southern Italy, the average percentage of serum prevalence in 2017 was 32.76% among a total of 16,627 tests, of which 5448 were positive, with a cutoff value of 1:160 [141].

Among the 30 species of *Leishmania* that can infect mammals, approximately 20 species can cause disease in humans [139]. The following main species are involved:

- *L. donovani complex* (which includes *L. donovani*, *L. infantum* and *L. chagasi*), which is responsible for VL (also called kala-azar). It causes irregular fever, hepatosplenomegaly, pancytopenia, and polyclonal hypergammaglobulinemia, with high mortality in untreated patients [138,142].
- *L. maior*, *L. tropica,* and *L. aethiopica* in the “Old World” and *L. braziliensis* and *L. mexicana* in the “New World” [143] cause CL. It causes painless persistent lesions that change from nodules to large ulcers, and can persist for months or years, leaving scars behind [138,142].
- *L. mexicana* and *L. braziliensis* cause ML, which affects nasopharyngeal tissues and can lead to extensive mutilation of the nose and palate [138,142].

VL is endemic in large areas of the tropics, subtropics, and Mediterranean basin [144]. Many infections are asymptomatic with a positive serological test, polymerase chain reaction (PCR), or leishmanin skin test in healthy individuals due to an immune response. The incubation period ranges from 10 days to one year [145], and patients present with insidious fever, weight loss, and organomegaly that persists for months [146]. Fever can be intermittent during the first period and then continuous [145]. The spleen and liver become massively enlarged, and lymphadenopathy can occur due to infection of the reticuloendothelial system. Parasites invading the bone marrow cause pancytopenia, which is responsible for pallor due to anemia; thrombocytopenia can cause hemorrhage; and leukopenia can be responsible for concurrent infections [145,147]. In the advanced stage, patients may become cachexic, and edematous due to hypoalbuminemia, and can present with heart failure due to anemia, jaundice, and ascites [146]. VL has emerged as an important opportunistic infection in endemic areas in the era of HIV infection [146]. In the presence of HIV coinfection, VL tends to be more severe and to manifest atypically, particularly in patients with advanced HIV disease [146]. HIV leads to a decrease in the number of T CD4 lymphocytes with a CD4 cell count usually below 200 cells/mL, and less than 100 cells/mL, and in 90% of these patients, the infection represents reactivation of a previously acquired subclinical or latent infection [148,149]. Although the initial presentations of VL are similar in HIV-infected and non-infected patients, HIV-coinfected patients have higher mortality rates, higher rates of initial treatment failure, and extremely high relapse rates, with the illness often having a prolonged chronic course [149]. Post-kala-azar leishmaniasis is a sequela of VL [150], which is characterized by hypopigmented macules, skin-colored nodules, and verrucous papules, predominantly affecting the face, but which can spread to the rest of the body. It occurs mainly in East Africa and India [150].

CL occurs in areas of the body exposed to insect bites, including the ears, nose, upper lip, cheeks, legs, hands, forearms, and ankles [148]. The initial lesion begins as an erythematous papule at the site of the bite and then develops into a painless nodule, which may produce pruritus [151]. Lesions may be solitary or multiple, and can spread through lymphatic vessels, causing lymphadenopathy [150]. The majority of the nodules have a seropurulent discharge that later dries and becomes a crust, and the removal of this crust reveals an ulcer/crater [152]. This “volcanic” nodule-ulcerative morphology is the most distinctive feature of acute cutaneous leishmaniosis (Figure 4) [143]. Usually, cutaneous leishmaniasis lesions are asymptomatic, unless secondary bacterial infections develop [143]. In addition, there is a diffuse form, presenting in the form of multiple papules and/or nodules, not ulcerated, which affects most of the skin [150].

ML is a chronic inflammatory process involving the nasal, pharyngeal, and laryngeal mucosa that can lead to extensive tissue destruction [153]; it appears in the form of ulcerated lesions that can be disfiguring [150].

In the case of coinfection with HIV, according to the WHO, the trend is an increase in manifestation even outside the endemic areas [142].

Co-infection results in a huge increase in the parasite in the blood of patients, which becomes a reservoir and increases the chances of spread. In this case, co-infection of HIV/*Leishmania* is considered a real risk factor in areas of Southwestern Europe [141]; among the 1700 cases recorded by the WHO from 33 countries, until 1998, 1440 occurred in this area. In particular, 885 cases were reported in Spain, 229 in Italy, 259 in France, and 117 in Portugal. Among the 960 cases, over 83% were men and over 85% were young adults between 20 and 40 years of age [141].

The diagnosis of CL should be considered in patients presenting with one or more chronic skin lesions and a history of exposure to an area where leishmaniasis is endemic. Diagnostic methods include visualization of the characteristic amastigote in smears or tissue (histopathology), parasite isolation through in vitro culture, and molecular detection of parasite DNA [154]. Adopting a multiple diagnostic approach, in collaboration with a specialized reference laboratory, is reasonable to enhance the likelihood of a positive result. Among available diagnostic methods, molecular amplification assays stand out as the most sensitive for diagnosis of CL [155,156]. In the context of diagnosing VL, demonstration of the parasite by smear or culture in tissue samples (typically bone marrow or spleen) is required. The appropriateness of employing less invasive diagnostic tools, such as identifying specific antibodies, antigens, or parasite DNA in peripheral blood specimens, varies depending on factors such as the patient’s clinical condition, parasite geographic origin, methodology employed, and laboratory experience [157]. For patients with HIV infection, serologic test sensitivity is compromised; in such cases, high parasite loads augment the sensitivity of culture and molecular assays in peripheral blood [154,158,159].

All pathogenic mechanisms play a complex role in *Leishmania* infection and are associated with both survival and death of the parasites, as well as with clinical evidence and diagnostic, prognostic, and therapeutic implications in the patient. There is also a correlation between the development of leishmaniasis and the immune system of a compromised host [160]. HIV-positive individuals are particularly susceptible to Leishmania infection and have a severe form of the disease. Indeed, the two infections work in synergy; VL accelerates the progression of HIV, while simultaneously, HIV makes it highly likely that asymptomatic *Leishmania* infection will turn symptomatic, thereby increasing the risk of VL in immunocompromised individuals [160].

During leishmaniasis infection, the Th1 response is associated with a protective effect, increasing IFN-γ production [161] and oxidative killing of intracellular amastigotes through reactive oxygen species (ROS) and nitric oxide (NO) release [162]. Conversely, a Th2 response is associated with susceptibility to infection, increasing IL-4 and IL-13 levels, and reducing intracellular parasite elimination due to high arginase-1 activity [57]. Therefore, there is a correlation between clinical manifestation and cytokine pathway response. Indeed, IL-4 and IL-13 (Th2 cytokines) can promote disease progression in CL, whereas only IL-4 appears to enhance protective type-1 responses in VL [57]. Several studies have reported that they play a dual role. Cytokines, such as IL-12, IL-7, and IFN-γ, can be involved in host protection, while others such as IL-9, IL-10, and TGF-β may be implicated in disease progression [161].

*Leishmania* infection in humans is usually subclinical, and the parasites may persist for the lifetime of the host, blocking, for example, the maturation of the complement system. It resists uptake by phagocytic DCs, delays phagolysosome formation [163], and blocks lysosomal proteolytic degradation [164]. *Leishmania* manipulates key aspects of host protection, involving several factors of the innate immune response, such as natural killer (NK) cells and mononuclear and polymorphonuclear phagocytes [165]. It also inhibits humoral immunity due to an increase of lymphocyte B-regulatory subpopulation CD19+/CD24^bright^/CD38^bright^ [166].

Activation of the TLR-9 receptor leads to increased IL-12 levels, stimulating NK cells to produce IFN-γ, which is involved in Th1 response [167]. The expression of IFNγ, TNFα [161], chemokine (C-C motif) ligand 2 (CCL2), CCL3, CCL11, and C-X-C motif chemokine ligand 10 (CXCL10) characterizes the immune response in situ, whereas CXCL9- and IFNγ-induced chemokines recruit T lymphocytes at the peripheral level [168]. This may explain the absence or low number of parasites in the lesions.

Furthermore, the parasite reduces the expression of CD40 T cells required for anti-parasitic activity and, implying Toll-like receptor TLR-2/TLR-4 activity, it inhibits the Janus tyrosine kinase (JAK)/signal transducer and activator of transcription (STAT) pathway in macrophages [165]. Therefore, it breaks off the cytokine cascade and normal expression of chemokines [165]. Consequently, targeting immune components such as macrophages is a potential method to combat infection and develop novel therapeutic strategies. In fact, macrophages control *Leishmania* replication through reactive oxygen species (ROS) and reactive nitrogen intermediates (RNIs) release and through interleukin IL-1β and IL-31 signaling [162].

As described by Dos Santos et al., the treatment of leishmaniasis with Bacillus Calmette-*Guerin* (BCG) vaccination in addition to β-glucan administration leads to protection against Leishmania and clinical improvement. Monocytes and macrophages induce non-specific protection against long-term secondary infection reprogramming through a process called “trained immunity,” which depends on metabolic and epigenetic changes. β-glucan-induced protection against *Leishmania* is mediated by IL-32 and IL-1 [162].

To date, several vaccination strategies have been studied in anti-Leishmania research, including formulations with live, attenuated, or killed organisms and antigen-based vaccines such as *Leishmania*-activated C-kinase antigen (LACK), as an immune activator or DNA-based vaccines. In particular, LACK antigens induced the production of protective cytokines in patients with active CL and VL, involving the production of Th1 cytokines (particularly IFN-γ and TNF-α) and granzyme B [169].

## 3. Future Perspectives and Conclusions

Mediterranean zoonoses remain largely unexplored in the field of infectious diseases. This narrative review reveals the ability of the pathogens to escape the host immune system, favoring its survival and proliferation. The immunological factors can be investigated for specific pharmacological targets and personalized therapy for the treatment of zoonoses. In this regard, multi-omics is an innovative approach that aims to identify molecular markers associated with biological processes across different “omic layers” (i.e., obtained from DNA, RNA, proteins, and metabolites); this combination of molecular biology techniques could lead to the discovery of predictive or prognostic biomarkers and new potential drug targets in the era of precision medicine [170]. Thus, the aim of applying multi-omics is to increase diagnostic tools by improving disease prognosis through a solid understanding of the genotype–phenotype relationship. Recently, some aspects of the life cycle of the etiological agents of Mediterranean zoonoses, such as the adaptability of some *Leishmania* strains [171] and the anti-apoptotic properties of proteins produced by *A. phagocytophilum* and *E. chaffeensis* [172], have been investigated. However, multi-omics techniques provide very detailed and complex information about a single microbiological strain, and the interactions between members of a community of microorganisms could remain obscured beneath the massive multi-omic datasets; therefore, the new techniques must be combined with the phenotypic techniques of classical microbiology to avoid ambiguity [173].

Furthermore, although there are important differences between Mediterranean zoonoses regarding their prevalence and incidence, they are characterized by common pathophysiological aspects and, consequently, similar clinical manifestations. Different microorganisms can cause common but non-specific syndromes (such as fever of unknown origin, vasculitis, and reticuloendothelial diseases). The biology and behavior of the vector are grafted into the complex balance between host and microorganism; therefore, in-depth knowledge cannot be separated from a “One Health” approach, which is indispensable for understanding and preventing this group of infectious diseases.

In conclusion, this paper focuses on selected diseases, providing the most important information that may be useful to clinicians. The importance of this review is supported by its finding that data on the incidence and prevalence of these diseases are outdated. The final aim of this paper is to improve the knowledge and understanding of these infections, to ensure clinicians are prepared for their re-emergence in the era of global warming. Clearly, diagnostic innovations and the availability of more accurate detection methods, together with better monitoring of the incidence and prevalence of these infections, are urgently needed to improve preparedness and response interventions.

## Figures and Tables

**Table 1:** Diagnostic criteria for Mediterranean Spotted Fever according to Raoult et al. (1992) [26].

Epidemiological Criteria	Points
Staying in an endemic area	**2**
Occurrence between May and September	**2**
Contact with dog ticks	**2**
**Clinical criteria**	
Fever > 39 °C	**5**
Eschar	**5**
Maculo-papular or purpuric rash	**5**
Two of the three clinical criteria	**3**
All three clinical criteria present	**5**
**Non-specific laboratory findings**	
Platelets < 150,000/mm^3^	**1**
ALT or AST > 50 u/L	**1**
**Bacteriological criteria**	
Isolation’s blood of *R*. *conorii*	**25**
*R. conorii* on immunofluorescence skin biopsy	**25**
**Serological criteria**	
Sole serum sample total Ig ≥ 1:128	**5**
Sole serum sample IgM ≥ 1:160 and IgG ≥ 1:128	**10**
Four-fold increase in paired serum samples	**20**

A score greater than 25 supports the diagnosis of Mediterranean Spotted Fever. ALT, alanine aminotransferase; AST, aspartate aminotransferase.

**Table 2:** Frequent complications of brucellosis [45].

Type of Complications	Complication	Comment
Skeletal	Arthritis Spondylitis Sacroiliitis Osteomyelitis Bursitis Tenosynovitis	Occur in approximately 20–85% of patients. In children, arthritis of the hip and knee joints is most common. Unilateral sacroiliitis is common in young adults. Spondylitis is the most serious complication and paraspinal and epidural abscess are not infrequent.
Neuro-psychological	Meningoencephalitis Cerebral abscess Myelitis neuritis Depression Psychosis	Occur in approximately 2–5% of patients. Cerebrospinal fluid examination reveals lymphocytic pleocytosis with elevated protein and normal or low glucose level. Gram’s stain and culture are of low sensitivity. Computerized tomography may demonstrate basal ganglia calcification and abscesses.
Genitourinary	Epididymo-orchitis Prostatitis and cystitis Interstitial nephritis Glomerulonephritis	Unilateral epididymo-orchitis is frequent in young men.
Cardiovascular	Endocarditis (aortic > mitral) Myocarditis and pericarditis Endarteritis	Occurs in <2% of cases, but is the most common cause of death. Embolic phenomena are common. Valve replacement is warranted in most cases. Mycotic aneurisms of the aorta and large vessels are rare.
Hepatobiliary	Non-granulomatous and granulomatous hepatitis Abscesses Cirrhosis Acute cholecystitis	Abnormal liver function tests occur in 30–90% of patients. Percutaneous drainage and prolonged course of antibiotics.
Spleen	Splenomegaly Spleen abscess Splenic calcifications	Surgical drainage of localized suppurative lesions and splenectomy may be of value if antimicrobial treatment is ineffective.
Pulmonary	Hilar adenopathy Perihilar infiltrates Nodular lesions Lung abscess Interstitial pattern and pleural effusions	Cough and other pulmonary symptoms in approximately 15–25% of patients.
Hematological	Anemia Leucopenia Thrombocytopenia and pancytopenia Disseminated intravascular coagulation	More common in patients with *Brucella melitensis*.
Cutaneous	Rashes Papules Petechiae Purpura Cutaneous granulomatous vasculitis and erythema nodosum	Occur in about 5% of patients. Many transient and often non-specific skin lesions have been described.
Others	Uveitis and thyroiditis	

**Table 3:** Virulence factors involved in Bartonellosis [80].

*Bartonella* Factor	Function	Pathogenesis
**TAAS (** **BadA, Vomps)**	Host cell binding, attachment to extracellular matrix, activation of HIF-1, auto-aggregation, activation of NF-κB pathway, and phagocytosis inhibition	Strong interaction with host cells favors the secretion of proangiogenic cytokines
***VirB/D4*** **IV** ***Bartonella*** **effector proteins**	Inhibition of apoptosis mechanism, proinflammatory and angiogenesis activation	Control of the intracellular defense mechanism
**OMP**	NF-κB pathway activation	Overexpression of E-selectin and ICAM-1 factors may be involved in proangiogenic pathway activation
**Deformin and Trw type IV secretion system**	Adhesion and membrane invaginations of erythrocytes	Erythrocyte invasion
**LPS**	Antagonizes the TLR4 pathway	Immunomodulation

TAAS, trimeric autotransporter adhesins; LPS, lipopolysaccharides; OMP, outer membrane proteins; ICAM, intercellular adhesion molecule; HIF, hypoxia-inducible factor.

**Table 4:** Primary clinical and laboratory manifestations of HGA and HME [84].

Symptom, Sign or Laboratory Finding	HME	HGA
	% (n), Pts	% (n), Pts
*Symptom or sign*		
Fever	97 (633)	93 (521)
Myalgia	57 (250)	77 (516)
Headache	80 (240)	76 (385)
Malaise	82 (234)	94 (288)
Nausea	64 (143)	38 (258)
Vomiting	33 (192)	26 (901)
Diarrhea	23 (197)	16 (951)
Cough	26 (155)	19 (260)
Arthralgias	41 (211)	46 (504)
Rash	31 (286)	6 (357)
Stiff neck	3 (240)	21 (24)
Confusion	19 (279)	17 (211)
*Laboratory finding*		
Leukopenia	62 (276)	49 (336)
Thrombocytopenia	71 (247)	71 (336)
Elevated serum AST or ALT level	83 (276)	71 (177)

HME, human monocytic ehrlichiosis; HGA, human granulocytic anaplasmosis; ALT, alanine aminotransferase; AST, aspartate aminotransferase; Pts, patients.

**Table 5:** Clinical complications and risk factors in human monocytic ehrlichiosis (HME) and human granulocytic anaplasmosis (HGA) [84].

Clinical Complication and Outcome	HME % Pts or Reports	HGA % Pts or Reports
*Hospitalization*	42	33–50
*Intensive care unit admission*	ND	7
*Life-threatening complication*	17	7
*Death*	3	<1
*Hemodynamic system complication* Toxic or septic-shock syndrome Coagulopathy Hemorrhage Myocarditis or heart failure Renal failure	++ 2 + + 2	+ <1 ± ± 1
*Pneumonia ARDS*	1	1
*CNS and PNS Complications* Meningoencephalitis Cranial nerve palsies Demyelinating polyneuropathy Brachial plexopathy	4 ± NR NR	1 ± ± ±
*Acute abdominal syndrome*	+	NR
*Rhabdomyolysis*	NR	±
*Opportunistic infection*	+	++

++, reported in >5 publications or cases; + reported several times; ± reported at least once; ARDS, acute respiratory distress syndrome; ND, not determined; NR, not reported; CNS, central nervous system; PNS, peripheral nervous system.

## Data Availability

Not applicable.

## References

[1] Alho A.M., Lima C., Colella V., de Carvalho L.M., Otranto D., Cardoso L. (2018). Awareness of zoonotic diseases and parasite control practices: A survey of dog and cat owners in Qatar. Parasites Vectors.

[2] Jánová E. (2019). Emerging and threatening vector-borne zoonoses in the world and in Europe: A brief update. Pathog. Glob Health..

[3] McArthur D.B. (2019). Emerging infectious diseases. Nurs. Clin..

[4] https://www.epicentro.iss.it/zecche/.

[5] Samuel G.H., Adelman Z.N., Myles K.M. (2016). Temperature-dependent effects on the replication and transmission of arthropod-borne viruses in their insect hosts. Curr. Opin. Insect Sci..

[6] Eisen R.J., Kugeler K.J., Eisen L., Beard C.B., Paddock C.D. (2017). Tick-Borne Zoonoses in the United States: Persistent and Emerging Threats to Human Health. ILAR J..

[7] Horka H., Staudt V., Klein M., Taube C., Reuter S., Dehzad N., Schmitt E. (2012). The tick salivary protein sialostatin L inhibits the Th9-derived production of the asthma-promoting cytokine IL-9 and is effective in the prevention of experimental asthma. J. Immunol..

[8] Sultana H., Neelakanta G. (2020). Arthropod exosomes as bubbles with message(s) to transmit vector-borne diseases. Curr. Opin. Insect Sci..

[9] Neelakanta G., Sultana H. (2022). Tick saliva and salivary glands: What do we know so far on their role in arthropod blood feeding and pathogen transmission. Front. Cell. Infect. Microbiol..

[10] Morelli A., Duranti A., Pauri P., Secondini S. (2016). Laboratory: A Privileged Point of View in Zoonoses Surveil-Lance. http://spvet.it/indice-spv.html#Numero99.

[11] Sgroi G., Iatta R., Lovreglio P., Stufano A., Laidoudi Y., Mendoza-Roldan J.A., Otranto D. (2022). Detection of Endosymbiont Candidatus Midichloria mitochondrii and tickborne pathogens in humans exposed to tick bites, Italy. Emerg. Infect. Dis..

[12] Osterloh A. (2017). Immune response against rickettsiae: Lessons from murine infection models. Med. Microbiol. Immunol..

[13] Milagres B.S., Gomes G.G., Galvao M.A.M., Freitas R.N., Pacheco R., Bouyer D.H., Montandon C.E., Silveira I., Barcelos R.M., Mafra C.L. (2010). Rickettsia in Synanthropic and Domestic Animals and Their Hosts from Two Areas of Low Endemicity for Brazilian Spotted Fever in the Eastern Region of Minas Gerais, Brazil. Am. J. Trop. Med. Hyg..

[14] https://www.epicentro.iss.it/zecche/FebbreBottonosa2001-2015.

[15] https://www.epicentro.iss.it/zecche/rickettsiosi.

[16] Heyman P., Cochez C., Hofhuis A., Van Der Giessen J., Sprong H., Porter S.R., Papa A. (2010). A clear and present danger: Tick-borne diseases in Europe. Exp. Rev. Anti-Infect. Ther..

[17] Creus B.F., Cueto F.B., Arenas E.E., Sanahuja R.V., Espin T.M., Salas M.N., Borrell A.C., Cuesta J.M., Porta F.S. (1985). Mediterranean Spotted Fever: A Cooperative Study of 227 Cases. Clin. Infect. Dis..

[18] Mert A., Ozaras R., Tabak F., Bilir M., Ozturk R. (2006). Mediterranean spotted fever: A review of fifteen cases. J. Dermatol..

[19] Rovery C., Brouqui P., Raoult D. (2008). Questions on Mediterranean Spotted Fever a Century after Its Discovery. Emerg. Infect. Dis..

[20] Parola P., Paddock C.D., Socolovschi C., Labruna M.B., Mediannikov O., Kernif T., Raoult D. (2013). Update on tick-borne rickettsioses around the world: A geographic approach. Clin. Microbiol. Rev..

[21] Faccini-Martínez A., García-Álvarez L., Hidalgo M., Oteo J.A. (2014). Syndromic classification of rickettsioses: An approach for clinical practice. Int. J. Infect. Dis..

[22] Chapman A.S. (2006). Diagnosis and management of tickborne rickettsial diseases: Rocky Mountain spotted fever, ehrlichioses, and anaplasmo-sis-United States: A practical guide for physicians and other health-care and public health professionals. MMWR Recomm. Rep..

[23] McQuiston J.H., Dunn J., Morris K., Carpenter L.R., Moncayo A.C., Chung I., McElroy K., Wiedeman C., Porter S., Kato C. (2014). Inadequacy of IgM Antibody Tests for Diagnosis of Rocky Mountain Spotted Fever. Am. J. Trop. Med. Hyg..

[24] La Scola B., Raoult D. (1997). Laboratory diagnosis of rickettsioses: Current approaches to diagnosis of old and new rickettsial diseases. J. Clin. Microbiol..

[25] Paris D.H., Dumler J.S. (2016). State of the art of diagnosis of rickettsial diseases: The use of blood specimens for diagnosis of scrub typhus, spotted fever group rickettsiosis, and murine typhus. Curr. Opin. Infect. Dis..

[26] Raoult D., Dupont H.T., Caraco P., Brouqui P., Drancourt M., Charrel C. (1992). Mediterranean spotted fever in Marseille: Descriptive epidemiology and the influence of climatic factors. Eur. J. Epidemiol..

[27] Silva-Ramos C.R., Hidalgo M., Faccini-Martínez A. (2021). Clinical, epidemiological, and laboratory features of Rickettsia parkeri rickettsiosis: A systematic review. Ticks Tick-Borne Dis..

[28] Helminiak L., Mishra S., Kim H.K. (2022). Pathogenicity and virulence of Rickettsia. Virulence.

[29] Papp S., Moderzynski K., Rauch J., Heine L., Kuehl S., Richardt U., Mueller H., Fleischer B., Osterloh A. (2016). Liver Necrosis and Lethal Systemic Inflammation in a Murine Model of Rickettsia typhi Infection: Role of Neutrophils, Macrophages and NK Cells. PLOS Neglected Trop. Dis..

[30] Janeway C.A., Travers P., Walport M., Shlomchik M.J. (2001). Immunobiology: The Immune System in Health and Disease. https://www.ncbi.nlm.nih.gov/books/NBK27100/.

[31] Ricklin D., Reis E.S., Lambris J.D. (2016). Complement in disease: A defence system turning offensive. Nat. Rev. Nephrol..

[32] Dahmani M., Cook J.H., Zhu J.C., Riley S.P. (2021). Contribution of classical complement activation and IgM to the control of *Rickettsia* infection. Mol. Microbiol..

[33] Klein M., Brühl T.-J., Staudt V., Reuter S., Grebe N., Gerlitzki B., Hoffmann M., Bohn T., Ulges A., Stergiou N. (2015). Tick Salivary Sialostatin L Represses the Initiation of Immune Responses by Targeting IRF4-Dependent Transcription in Murine Mast Cells. J. Immunol..

[34] Koch S., Sopel N., Finotto S. (2017). Th9 and other IL-9-producing cells in allergic asthma. Semin. Immunopathol..

[35] Tete S., Saggini A., Maccauro G., Rosati M., Conti F., Cianchetti E., Tripodi D., Toniato E., Fulcheri M., Salini V. (2012). Interleukin-9 and mast cells. J. Biol. Regul. Homeost. Agents.

[36] https://www.epicentro.iss.it/brucellosi/epidemiologia-italia.

[37] Godfroid J., Cloeckaert A., Liautard J.P., Kohler S., Fretin D., Walravens K., Letesson J.J. (2005). From the discovery of the Malta fever’s agent to the discovery of a marine mammal reservoir, brucellosis has continuously been a re-emerging zoonosis. Vet. Res..

[38] Seleem M., Boyle S.M., Sriranganathan N. (2010). Brucellosis: A re-emerging zoonosis. Veter. Microbiol..

[39] https://www.uptodate.com/contents/brucellosis-epidemiology-microbiology-clinical-manifestations-and-diagnosis.

[40] https://www.ecdc.europa.eu/en/brucellosis.

[41] Pappas G., Aritidis N., Bosilovsi M., Tsianos E. (2005). Brucellosis. N. Engl. J. Med..

[42] Harrison E.R., Posada R. (2018). Brucellosis. Pediatr. Rev..

[43] Bukhari E. (2018). Pediatric brucellosis: An update review for the new millennium. Saudi Med. J..

[44] Franco M.P., Mulder M., Gilman R.H., Smits H.L. (2007). Human brucellosis. Lancet Infect. Dis..

[45] Solera J., Martinez-Alfaro E., Espinosa A. (1997). Recognition and Optimum Treatment of Brucellosis. Drugs.

[46] Brucellosis Reference Guide Centers for Disease Control (CDC). https://www.cdc.gov/brucellosis/pdf/brucellosi-reference-guide.pdf.

[47] Al Dahouk S., Tomaso H., Nöckler K., Neubauer H., Frangoulidis D. (2003). Laboratory-based diagnosis of brucellosis—A review of the literature. Part II: Serological tests for brucellosis. Clin. Lab..

[48] de los Ángeles Mantecón M., Gutiérrez M.P., del Pilar Zarzosa M., Fernández-Lago L., de Dios Colmenero J., Vizcaíno N., Bratos M.A., Almaraz A., Cubero A., Muñoz M.F. (2008). Influence of brucellosis history on serological diagnosis and evolution of patients with acute brucellosis. J. Infect..

[49] Yagupsky P., Morata P., Colmenero J.D. (2019). Laboratory Diagnosis of Human Brucellosis. Clin. Microbiol. Rev..

[50] López-Santiago R., Sánchez-Argáez A.B., De Alba-Núñez L.G., Baltierra-Uribe S.L., Moreno-Lafont M.C. (2019). Immune Response to Mucosal Brucella Infection. Front. Immunol..

[51] Muñoz González F., Sycz G., Alonso Paiva I.M., Linke D., Zorreguieta A., Baldi P.C., Ferrero M.C. (2019). The BtaF adhesin is necessary for full virulence during respiratory infection by Brucella suis and is a novel immunogen for nasal vaccination against Brucella infection. Front. Immunol..

[52] Bialer M.G., Sycz G., González F.M., Ferrero M.C., Baldi P.C., Zorreguieta A. (2020). Adhesins of *Brucella*: Their Roles in the Interaction with the Host. Pathogens.

[53] Ferrero M.C., Fossati C.A., Rumbo M., Baldi P.C. (2012). Brucella invasion of human intestinal epithelial cells elicits a weak proinflammatory response but a significant CCL20 secretion. FEMS Immunol. Med. Microbiol..

[54] Paixao T.A., Roux C.M., den Hartigh A.B., Sankaran-Walters S., Dandekar S., Santos R.L., Tsolis R.M. (2009). Establishment of systemic Brucella melitensis infection through the digestive tract requires urease, the type IV secretion system, and lipopolysaccharide O antigen. Infect. Immun..

[55] Copin R., Vitry M.-A., Mambres D.H., Machelart A., De Trez C., Vanderwinden J.-M., Magez S., Akira S., Ryffel B., Carlier Y. (2012). In Situ Microscopy Analysis Reveals Local Innate Immune Response Developed around Brucella Infected Cells in Resistant and Susceptible Mice. PLOS Pathog..

[56] Vitry M.A., Hanot Mambres D., De Trez C., Akira S., Ryffel B., Letesson J.J., Muraille E. (2014). Humoral immunity and CD4+ Th1 cells are both necessary for a fully protective immune response upon secondary infection with Brucella melitensis. J. Immunol..

[57] Ives A., Masina S., Castiglioni P., Prevel F., Revaz-Breton M., Hartley M.A., Ronet C. (2014). MyD88 and TLR9 dependent immune responses mediate resistance to Leishmania guyanensis infections, irrespective of Leishmania RNA virus burden. PLoS ONE.

[58] Deqiu S., Donglou X., Jiming Y. (2002). Epidemiology and control of brucellosis in China. Veter Microbiol..

[59] Abkar M., Fasihi-Ramandi M., Kooshki H., Lotfi A.S. (2018). Intraperitoneal immunization with Urease loaded N-trimethyl Chitosan nanoparticles elicits high protection against Brucella melitensis and Brucella abortus infections. Immunol. Lett..

[60] Mazzitelli M., Lamberti A.G., Quirino A., Marascio N., Barreca G.S., Costa C., Torti C. (2018). Utility of molecular identification and quantitation of Bartonella species with species-specific real-time PCR for monitoring treatment response: A case series. Open Microbiol. J..

[61] Mosbacher M.E., Klotz S., Klotz J., Pinnas J.L. (2011). Bartonella henselae and the potential for arthropod vector-borne transmission. Vector-Borne Zoonot. Dis..

[62] Chomel B.B., Boulouis H.-J., Maruyama S., Breitschwerdt E.B. (2006). *Bartonella* Spp. in Pets and Effect on Human Health. Emerg. Infect. Dis..

[63] Graziani C., Duranti A., Morelli A., Busani L., Pezzotti P. Zoonosi in Italia nel Periodo 2009–2013. Roma: Istituto Superiore di Sanità. 2016. 2016. Rapporti ISTISAN 16/1. https://www.iss.it/rapporti-istisan/-/asset_publisher/Ga8fOpve0fNN/content/id/5183699.

[64] Bos F., Chauveau B., Ruel J., Fontant G., Campistron E., Meunier C., Jambon F., Moreau K., Delmas Y., Couzi L. (2022). Serious and Atypical Presentations of *Bartonella henselae* Infection in Kidney Transplant Recipients. Open Forum Infect. Dis..

[65] Luciani L., El Baroudi Y., Prudent E., Raoult D., Fournier P.-E. (2021). Bartonella infections diagnosed in the French reference center, 2014–2019, and focus on infections in the immunocompromised. Eur. J. Clin. Microbiol. Infect. Dis..

[66] Mosepele M., Mazo D., Cohn J. (2011). *Bartonella* Infection in Immunocompromised Hosts: Immunology of Vascular Infection and Vasoproliferation. J. Immunol. Res..

[67] Karem K.L., Paddock C.D., Regnery R.L. (2000). Bartonella henselae, B. quintana, and B. bacilliformis: Historical pathogens of emerging significance. Microbes Infect..

[68] Raoult D., Roux V. (1999). The Body Louse as a Vector of Reemerging Human Diseases. Clin. Infect. Dis..

[69] Foucault C., Brouqui P., Raoult D. (2006). Bartonella quintana Characteristics and Clinical Management. Emerg Infect Dis..

[70] Maurin M., Raoult D. (1996). Bartonella (Rochalimaea) quintana infections. Clin. Microbiol. Rev..

[71] https://www.uptodate.com/contents/clinical-features-diagnosis-and-treatment-of-bartonella-quintana-infections.

[72] Ohl M.E., Spach D.H. (2000). Bartonella quintana and Urban Trench Fever. Clin. Infect. Dis..

[73] Spach D.H., Koehler J.E. (1998). Bartonella-associated infections. Infect. Dis. Clin..

[74] Jacomo V., Kelly P.J., Raoult D. (2002). Natural History of *Bartonella* Infections (an Exception to Koch’s Postulate). Clin. Vaccine Immunol..

[75] Carithers H.A. (1985). Cat-scratch disease: An overview based on a study of 1200 patients. Am. J. Dis. Child..

[76] Charles R.C., Sertic M., Neilan A.M., Sohani A.R. (2021). Case 11-2021: A 39-year-old woman with fever, flank pain, and inguinal lymphadenopathy. N. Engl. J. Med..

[77] Paddock C.D., Childs J.E. (2003). *Ehrlichia chaffeensis*: A Prototypical Emerging Pathogen. Clin. Microbiol. Rev..

[78] Angelakis E., Raoult D. (2014). Pathogenicity and treatment of Bartonella infections. Int. J. Antimicrob. Agents.

[79] Choi E.W., Lee H.W., Lee J.S., Kim I.Y., Shin J.H., Seong J.K. (2019). Ahnak-knockout mice show susceptibility to Bartonella henselae infection because of CD4+ T cell inactivation and decreased cytokine secretion. BMB Rep..

[80] Harms A., Dehio C. (2012). Intruders below the Radar: Molecular Pathogenesis of *Bartonella* spp.. Clin. Microbiol. Rev..

[81] Matza D., Badou A., Kobayashi K.S., Goldsmith-Pestana K., Masuda Y., Komuro A., McMahon-Pratt D., Marchesi V.T., Flavell R.A. (2008). A Scaffold Protein, AHNAK1, Is Required for Calcium Signaling during T Cell Activation. Immunity.

[82] https://www.uptodate.com/contents/human-ehrlichiosis-and-anaplasmosis.

[83] Dumler J.S., Choi K.-S., Garcia-Garcia J.C., Barat N.S., Scorpio D.G., Garyu J.W., Grab D.J., Bakken J.S. (2005). Human Granulocytic Anaplasmosis and *Anaplasma phagocytophilum*. Emerg. Infect. Dis..

[84] Dumler J.S., Madigan J.E., Pusterla N., Bakken J.S. (2007). Ehrlichioses in Humans: Epidemiology, Clinical Presentation, Diagnosis, and Treatment. Clin. Infect. Dis..

[85] Guccione C., Colomba C., Tolomeo M., Trizzino M., Iaria C., Cascio A. (2021). Rickettsiales in Italy. Pathogens.

[86] Heitman K.N., Dahlgren F.S., Drexler N.A., Massung R.F., Behravesh C.B. (2016). Increasing incidence of ehrlichiosis in the United States: A summary of national surveillance of Ehrlichia chaffeensis and Ehrlichia ewingii infections in the United States, 2008–2012. Am. J. Trop. Med. Hyg..

[87] Moniuszko-Malinowska A., Dunaj J., Andersson M.O., Czupryna P., Zajkowska J., Guziejko K., Pancewicz S. (2020). Assessment of Anaplasma phagocytophilum presence in early Lyme borreliosis manifested by erythema migrans skin lesions. Travel Med. Infect. Dis..

[88] Moniuszko-Malinowska A., Dunaj J., Andersson M.O., Chmielewski T., Czupryna P., Groth M., Pancewicz S. (2021). Anaplasmosis in Poland–analysis of 120 patients. Ticks Tick-borne Dis..

[89] Ehrlichiosis and Anaplasmosis 2008 Case Definition. United States Centers for Disease Control. https://ndc.services.cdc.gov/case-definitions/ehrlichiosis-and-anaplasmosis-2008/.

[90] Blanco J., Oteo J. (2002). Human granulocytic ehrlichiosis in Europe. Clin. Microbiol. Infect..

[91] Bakken J.S., Dumler J.S. (2006). Clinical diagnosis and treatment of human granulocytotropic anaplasmosis. Ann. N. Y. Acad. Sci..

[92] Bakken J.S., Dumler J.S. (2015). Human granulocytic anaplasmosis. Infect. Dis. Clin..

[93] Buller R.S., Arens M., Hmiel S.P., Paddock C.D., Sumner J.W., Rikihisa Y., Unver A., Gaudreault-Keener M., Manian F.A., Liddell A.M. (1999). *Ehrlichia ewingii*, a Newly Recognized Agent of Human Ehrlichiosis. N. Engl. J. Med..

[94] Chen S., Yu X., Popov V.L., Westerman E.L., Hamilton F.G., Walker D.H. (1997). Genetic and Antigenic Diversity of *Ehrlichia chaffeensis*: Comparative Analysis of a Novel Human Strain from Oklahoma and Previously Isolated Strains. J. Infect. Dis..

[95] Chapes S.K., Ganta R.R. (2008). Defining the immune response to Ehrlichia species using murine models. Vet. Parasitol..

[96] Jankovic D., Kullberg M.C., Hieny S., Caspar P., Collazo C.M., Sher A. (2002). In the absence of IL-12, CD4^+^ T cell responses to intracellular pathogens fail to default to a Th2 pattern and are host protective in an IL-10^−/−^ setting. Immunity.

[97] Kuriakose J.A., Zhang X., Luo T., McBride J.W. (2012). Molecular basis of antibody mediated immunity against Ehrlichia chaffeensis involves species-specific linear epitopes in tandem repeat proteins. Microbes Infect..

[98] Velayutham T.S., Kumar S., Zhang X., Kose N., Walker D.H., Winslow G., Crowe J.E., McBride J.W. (2019). Ehrlichia chaffeensis Outer Membrane Protein 1-Specific Human Antibody-Mediated Immunity Is Defined by Intracellular TRIM21-Dependent Innate Immune Activation and Extracellular Neutralization. Infect. Immun..

[99] https://www.uptodate.com/contents/treatment-of-lyme-disease.

[100] Verhaegh D., Joosten L.A., Oosting M. (2017). The role of host immune cells and Borrelia burgdorferi antigens in the etiology of Lyme disease. Eur. Cytok. Netw..

[101] https://www.epicentro.iss.it/zecche/borreliosi.

[102] Steere A.C., Malawista S.E., Snydman D.R., Shope R.E., Andiman W.A., Ross M.R., Steele F.M. (1977). Lyme Arthritis: An Epidemic of Oligoarticular Arthritis in Children and Adults in Three Connecticut Communities. Arthr. Rheumatol. Eur. J. Epidemiol..

[103] Benach J.L., Bosler E.M., Hanrahan J.P., Coleman J.L., Habicht G.S., Bast T.F., Cameron D.J., Ziegler J.L., Barbour A.G., Burgdorfer W. (1983). Spirochetes Isolated from the Blood of Two Patients with Lyme Disease. N. Engl. J. Med..

[104] Burgdorfer W., Barbour A.G., Hayes S.F., Benach J.L., Grunwaldt E., Davis J.P. (1982). Lyme Disease—A Tick-Borne Spirochetosis?. Science.

[105] Rizzoli A., Hauffe H.C., Carpi G., Vourc’h G.I., Neteler M., Rosà R. (2011). Lyme borreliosis in Europe. Eurosurveillance.

[106] Vandekerckhove O., De Buck E., Van Wijngaerden E. (2019). Lyme disease in Western Europe: An emerging problem? A systematic review. Acta Clin. Belg..

[107] D’Amico W., De Merich D., Di Renzi S., D’Ovidio M.C., Martini A., Melis P., Tomao P., Vonesch N. (2018). Dipartimento di Medicina, Epidemiologia, Igiene del Lavoro e Ambientale. Inail. https://dsv.units.it/sites/dsv.units.it/files/Zoonosi-trasmesse-da-zecche_documento%20INAIL_12_07_2018_conEC.pdf.

[108] Sykes R.A., Makiello P. (2017). An estimate of Lyme borreliosis incidence in Western Europe. J. Public Health.

[109] Beltrame A., Rodari P., Mauroner L., Zanella F., Moro L., Bertoli G., Da Re F., Russo F., Napoletano G., Silva R. (2020). Emergence of Lyme borreliosis in the province of Verona, Northern Italy: Five-years of sentinel surveillance. Ticks Tick-borne Dis..

[110] Cook M.J. (2014). Lyme borreliosis: A review of data on transmission time after tick attachment. Int. J. Gen. Med..

[111] Piesman J., Gern L. (2004). Lyme borreliosis in Europe and North America. Parasitology.

[112] Stanek G., Strle F. (2018). Lyme borreliosis–from tick bite to diagnosis and treatment. FEMS Microbiol. Rev..

[113] Steere A.C. (2001). Lyme Disease. N. Engl. J. Med..

[114] Steere A.C., Dhar A., Hernandez J., Fischer P.A., Sikand V.K., Schoen R.T., Nowakowski J., McHugh G., Persing D.H. (2003). Systemic symptoms without erythema migrans as the presenting picture of early Lyme disease. Am. J. Med..

[115] Branda J.A., Steere A.C. (2021). Laboratory diagnosis of Lyme borreliosis. Clin. Microbiol. Rev..

[116] Aguero-Rosenfeld M.E., Wang G., Schwartz I., Wormser G.P. (2005). Diagnosis of Lyme Borreliosis. Clin. Microbiol. Rev..

[117] Bradley J.F., Johnson R.C., Goodman J.L. (1994). The Persistence of Spirochetal Nucleic Acids in Active Lyme Arthritis. Ann. Intern. Med..

[118] Nocton J.J., Dressler F., Rutledge B.J., Rys P.N., Persing D.H., Steere A.C. (1994). Detection of Borrelia burgdorferi DNA by Polymerase Chain Reaction in Synovial Fluid from Patients with Lyme Arthritis. N. Engl. J. Med..

[119] Sigal L.H. (1994). The polymerase chain reaction assay for Borrelia burgdorferi in the diagnosis of Lyme disease. Ann. Int. Med..

[120] Nocton J.J., Bloom B.J., Rutledge B.J., Persing D.H., Logigian E.L., Schmid C.H., Steere A.C. (1996). Detection of Borrelia burgdorferi DNA by Polymerase Chain Reaction in Cerebrospinal Fluid in Lyme Neuroborreliosis. J. Infect. Dis..

[121] Guérin M., Shawky M., Zedan A., Octave S., Avalle B., Maffucci I., Padiolleau-Lefèvre S. (2023). Lyme borreliosis diagnosis: State of the art of improvements and innovations. BMC Microbiol..

[122] Callister S.M., Jobe D.A., Stuparic-Stancic A., Miyamasu M., Boyle J., Dattwyler R.J., Arnaboldi P.M. (2016). Detection of IFN-γ secretion by T cells collected before and after successful treatment of early Lyme disease. Clin. Infect. Dis..

[123] Lintner H., Hochgatterer-Rechberger P., Pischinger B., Seier J., Vollmann P., Haushofer A., Topakian R. (2020). Sensitivity and specificity of cerebrospinal fluid CXCL13 for diagnosing Lyme neuroborreliosis-a study on 1410 patients and review of the literature. J. Neurol. Sci..

[124] Pilz G., Steger R., Wipfler P., Otto F., Afazel S., Haschke-Becher E., Harrer A. (2020). Beyond LNB: Real life data on occurrence and extent of CSF CXCL13 in neuroinflammatory diseases. J. Neuroimmunol..

[125] Molins C.R., Ashton L.V., Wormser G.P., Hess A.M., Delorey M.J., Mahapatra S., Belisle J.T. (2015). Development of a metabolic biosignature for detection of early Lyme disease. Clin. Infect. Dis..

[126] Branda J.A., Lemieux J.E., Blair L., Ahmed A.A., Hong D.K., Bercovici S., Pollock N.R. (2021). Detection of Borrelia burgdorferi cell-free DNA in human plasma samples for improved diagnosis of early Lyme borreliosis. Clin. Infect. Dis..

[127] Van de Schoor F.R., Baarsma M.E., Gauw S.A., Kullberg B.J., van den Wijngaard C.C., Hovius J.W. (2019). Validation of cellular tests for Lyme borreliosis (VICTORY) study. BMC Infect. Dis..

[128] Samuels J.D.R., Scott D. (2021). Lyme Disease and Relapsing Fever Spirochetes: Genomics, Molecular Biology, Host Interactions and Disease Pathogenesis.

[129] Wormser G.P., McKenna D., Carlin J., Nadelman R.B., Cavaliere L.F., Holmgren D., Byrne D.W., Nowakowski J. (2005). Brief Communication: Hematogenous Dissemination in Early Lyme Disease. Ann. Intern. Med..

[130] Robin J., Bluth M., Ruditzky M., Norowitz K., Chice S., Pytlak E., Nowakowski M., Durkin H., Smith-Norowitz T. (2005). IgE anti-Borrelia Burgdorferi components (p18, p31, p34, p41, p45, p60) and increased blood CD8+CD60+ T cells in children with lyme disease. J. Allergy Clin. Immunol..

[131] Gomes-Solecki M., Arnaboldi P.M., Backenson P.B., Benach J.L., Cooper C.L., Dattwyler R.J., Schutzer S.E., Marconi R.T., Marques A.R., Molloy P. (2020). Protective immunity and new vaccines for Lyme disease. Clin. Infect. Dis..

[132] Gomes-Solecki M. (2014). Blocking pathogen transmission at the source: Reservoir targeted OspA-based vaccines against Borrelia burgdorferi. Front. Cell. Infect. Microbiol..

[133] Klouwens M.J., Trentelman J.J.A., Wagemakers A., Ersoz J.I., Bins A.D., Hovius J.W. (2021). Tick-Tattoo: DNA Vaccination against B. burgdorferi or Ixodes scapularis Tick Proteins. Front. Immunol..

[134] Kaye P., Scott P. (2011). Leishmaniasis: Complexity at the host–pathogen interface. Nat. Rev. Genet..

[135] https://www.cdc.gov/parasites/leishmaniasis/index.html.

[136] https://www.salute.gov.it/portale/sanitaAnimale/dettaglioContenutiSanitaAnimale.jsp?lingua=italiano&id=220&tab=1.

[137] https://www.izsvenezie.it/temi/malattie-patogeni/leishmaniosi/.

[138] Cecílio P., Cordeiro-Da-Silva A., Oliveira F. (2022). Sand flies: Basic information on the vectors of leishmaniasis and their interactions with Leishmania parasites. Commun. Biol..

[139] World Health Organization Leishmaniasis. https://www.who.int/health-topics/leishmaniasis#tab=tab_1.

[140] https://www.epicentro.iss.it/leishmaniosi/epidemiologia.

[141] https://www.salute.gov.it/portale/sanitaAnimale/dettaglioContenutiSanitaAnimale.jsp?lingua=italiano&id=220&tab=2.

[142] https://www.who.int/news/item/08-06-2022-visceral-leishmaniasis-and-HIV-coinfection-WHO-publishes-new-guideline-with-region-specific-treatment-recommendations.

[143] Salman S.M., Rubeiz N.G., Kibbi A.-G. (1999). Cutaneous leishmaniasis: Clinical features and diagnosis. Clin. Dermatol..

[144] Srivastava P., Dayama A., Mehrotra S., Sundar S. (2011). Diagnosis of visceral leishmaniasis. Transact. R. Soc. Trop. Med. Hyg..

[145] Saporito L., Giammanco G.M., De Grazia S., Colomba C. (2013). Visceral leishmaniasis: Host–parasite interactions and clinical presentation in the immunocompetent and in the immunocompromised host. Int. J. Infect. Dis..

[146] Van Griensven J., Diro E. (2012). Visceral leishmaniasis. Infect. Dis. Clin..

[147] Wilson M.E., Streit J.A. (1996). Visceral leishmaniasis. Gastroenterol. Clin..

[148] Torres-Guerrero E., Quintanilla-Cedillo M.R., Ruiz-Esmenjaud J., Arenas R. (2017). Leishmaniasis: A review. F1000Research.

[149] Jarvis J.N., Lockwood D.N. (2013). Clinical aspects of visceral leishmaniasis in HIV infection. Curr. Opin. Infect. Dis..

[150] Abadías-Granado I., Diago A., Cerro P.A., Palma-Ruiz A.M., Gilaberte Y. (2021). Leishmaniasis cutánea y mucocutánea. Actas Dermo-Sifiliográfic..

[151] Dowlati Y. (1996). Cutaneous leishmaniasis: Clinical aspect. Clin. Dermatol..

[152] Kubba R., Al-Gindan Y., El-Hassan A., Omer A. (1987). Clinical diagnosis of cutaneous leishmaniasis (oriental sore). J. Am. Acad. Dermatol..

[153] Ahluwalia S. (2004). Mucocutaneous leishmaniasis: An imported infection among travellers to central and South America. BMJ.

[154] Aronson N., Herwaldt B.L., Libman M., Pearson R., Lopez-Velez R., Weina P., Magill A. (2016). Diagnosis and treatment of leishmaniasis: Clinical practice guidelines by the Infectious Diseases Society of America (IDSA) and the American Society of Tropical Medicine and Hygiene (ASTMH). Clin. Infect. Dis..

[155] Boggild A.K., Ramos A.P., Espinosa D., Valencia B.M., Veland N., Miranda-Verastegui C., Llanos-Cuentas A. (2010). Clinical and demographic stratification of test performance: A pooled analysis of five laboratory diagnostic methods for American cutaneous leishmaniasis. Am. J. Trop. Med. Hyg..

[156] Reithinger R., Dujardin J.C. (2007). Molecular diagnosis of leishmaniasis: Current status and future applications. J. Clin. Microbiol..

[157] Sundar S., Rai M. (2002). Laboratory diagnosis of visceral leishmaniasis. Clin. Vaccine Immunol..

[158] Alvar J., Aparicio P., Aseffa A., Den Boer M., Canavate C., Dedet J.P., Moreno J. (2008). The relationship between leishmaniasis and AIDS: The second 10 years. Clin. Microbiol. Rev..

[159] van Griensven J., Zijlstra E.E., Hailu A. (2014). Visceral Leishmaniasis and HIV Coinfection: Time for Concerted Action. PLOS Neglected Trop. Dis..

[160] Burki T. (2022). Guidelines for visceral leishmaniasis and HIV co-infection. Lancet Infect. Dis..

[161] Dayakar A., Chandrasekaran S., Kuchipudi S.V., Kalangi S.K. (2019). Cytokines: Key determinants of resistance or disease progression in visceral leishmaniasis: Opportunities for novel diagnostics and immunotherapy. Front. Immunol..

[162] Dos Santos J.C., de Figueiredo A.M.B., Silva M.V.T., Cirovic B., de Bree L.C.J., Damen M.S., Joosten L.A. (2019). β-Glucan-induced trained immunity protects against Leishmania braziliensis infection: A crucial role for IL-32. Cell Rep..

[163] Gueirard P., Laplante A., Rondeau C., Milon G., Desjardins M. (2008). Trafficking of Leishmania donovani promastigotes in non-lytic compartments in neutrophils enables the subsequent transfer of parasites to macrophages. Cell. Microbiol..

[164] Laufs H., Müller K., Fleischer J., Reiling N., Jahnke N., Jensenius J.C., Laskay T. (2002). Intracellular survival of Leishmania major in neutrophil granulocytes after uptake in the absence of heat-labile serum factors. Infect. Immun..

[165] Hawn T.R., Ozinsky A., Underhill D.M., Buckner F.S., Akira S., Aderem A. (2002). Leishmania major activates IL-1α expression in macrophages through a MyD88-dependent pathway. Microbes Infect..

[166] Matera G., Torti C., Mazzitelli M., Greco G., Rania A., Peronace C., Settembre P., Galati L., Giancotti A., Lamberti A.G. (2018). Depression of lymphocyte activity during cutaneous leishmaniasis: A case report. Diagn. Microbiol. Infect. Dis..

[167] Liese J., Schleicher U., Bogdan C. (2008). The innate immune response against Leishmania parasites. Immunobiology.

[168] Machado G.U., Prates F.V., Machado P.R.L. (2019). Disseminated leishmaniasis: Clinical, pathogenic, and therapeutic aspects. An. Bras. Dermatol..

[169] Fernández L., Carrillo E., Sánchez-Sampedro L., Sánchez C., Ibarra-Meneses A.V., Jimenez M.A., Moreno J. (2018). Antigenicity of leishmania-activated C-kinase antigen (LACK) in human peripheral blood mononuclear cells, and protective effect of prime-boost vaccination with pCI-neo-LACK plus attenuated LACK-expressing Vaccinia viruses in hamsters. Front. Immunol..

[170] Krassowski M., Das V., Sahu S.K., Misra B.B. (2020). State of the Field in Multi-Omics Research: From Computational Needs to Data Mining and Sharing. Front. Genet..

[171] Cuypers B., Meysman P., Erb I., Bittremieux W., Valkenborg D., Baggerman G., Mertens I., Sundar S., Khanal B., Notredame C. (2022). Four layer multi-omics reveals molecular responses to aneuploidy in Leishmania. PLOS Pathog..

[172] Li R., Ma Z., Zheng W., Wang Z., Yi J., Xiao Y., Chen C. (2022). Multiomics analyses reveals Anaplasma phagocytophilum Ats-1 induces anti-apoptosis and energy metabolism by upregulating the respiratory chain-mPTP axis in eukaryotic mitochondria. BMC Microbiol..

[173] Vilanova C., Porcar M. (2016). Are multi-omics enough?. Nat. Microbiol..

